# Amyloid beta and its naturally occurring N-terminal variants are potent activators of human and mouse formyl peptide receptor 1

**DOI:** 10.1016/j.jbc.2022.102642

**Published:** 2022-10-27

**Authors:** Lukas Busch, Zukaa al Taleb, Yu-Liang Tsai, Vu Thu Thuy Nguyen, Qi Lu, Christopher V. Synatschke, Kristina Endres, Bernd Bufe

**Affiliations:** 1 Department of Informatics and Microsystems Technology, University of Applied Sciences Kaiserslautern, Zweibrücken, Germany; 2 Department Synthesis of Macromolecules, Max Planck Institute for Polymer Research, Mainz, Germany; 3 Department of Psychiatry and Psychotherapy, University Medical Centre of the Johannes Gutenberg-University, Mainz, Germany

**Keywords:** formyl peptide receptors, neuroinflammation, amyloid beta, Alzheimer's disease, glia, AD, Alzheimer's disease, APP, amyloid precursor protein, Aβ, amyloid beta, DMSO, dimethyl sulfoxide, FCS, fetal calf serum, FPR, Formyl peptide receptor, HFIP, Hexafluoroisopropanol, PDL, poly-D-lysine, PRR, pattern recognition receptor, TEM, transmission electron microscopy, ThT, thioflavin T

## Abstract

Formyl peptide receptors (FPRs) may contribute to inflammation in Alzheimer’s disease through interactions with neuropathological Amyloid beta (Aβ) peptides. Previous studies reported activation of FPR2 by Aβ_1-42_, but further investigation of other FPRs and Aβ variants is needed. This study provides a comprehensive overview of the interactions of mouse and human FPRs with different physiologically relevant Aβ-peptides using transiently transfected cells in combination with calcium imaging. We observed that, in addition to hFPR2, all other hFPRs also responded to Aβ_1-42_, Aβ_1-40_, and the naturally occurring variants Aβ_11-40_ and Aβ_17-40_. Notably, Aβ_11-40_ and Aβ_17-40_ are very potent activators of mouse and human FPR1, acting at nanomolar concentrations. Buffer composition and aggregation state are extremely crucial factors that critically affect the interaction of Aβ with different FPR subtypes. To investigate the physiological relevance of these findings, we examined the effects of Aβ_11-40_ and Aβ_17-40_ on the human glial cell line U87. Both peptides induced a strong calcium flux at concentrations that are very similar to those obtained in experiments for hFPR1 in HEK cells. Further immunocytochemistry, qPCR, and pharmacological experiments verified that these responses were primarily mediated through hFPR1. Chemotaxis experiments revealed that Aβ_11-40_ but not Aβ_17-40_ evoked cell migration, which argues for a functional selectivity of different Aβ peptides. Together, these findings provide the first evidence that not only hFPR2 but also hFPR1 and hFPR3 may contribute to neuroinflammation in Alzheimer’s disease through an interaction with different Aβ variants.

Alzheimer’s disease (AD) is a complex neurodegenerative disorder with a heterogeneous pathobiology leading to progressive dementia with death as an inevitable outcome, generally within 5 to 12 years after symptom onset (1). While there is an urgent need for therapies that may prevent or slow the progression of AD, no unequivocal treatment is currently available (2). A better understanding of the molecular mechanisms underlying AD can help to identify new strategies to develop such treatments. The discovery that extracellular amyloid beta (Aβ) depositions (3) and intracellular accumulation of hyperphosphorylated tau (4, 5) are common neuropathological hallmarks of all AD forms were significant advances that helped to clarify several key aspects of the underlying pathology. Although these discoveries are already more than 3 decades old, the precise impact of Aβ on neuroinflammation and neurodegeneration is still incompletely understood (2). Major challenges in Aβ-research comprise the complex peptide processing involving multiple proteases (6), the challenging physiochemical properties of the resulting fragments that permit a formation of different oligomeric structures (7), and the pleiotropic physiological effects of Aβ-peptides on neurons, glia, and immune cells (8, 9).

Aβ is produced by a sequential cleavage of an amyloid precursor protein (APP) by β- and γ-secretase (10). Cleavage can occur at several sites, which results in the predominant production of peptides ending at position 38, 40, or 42 of the Aβ domain (10, 11). APP processing is somewhat imprecise and thus, depending on the specific (patho)physiological conditions, several additional longer and shorter Aβ variants in varying concentrations can occur (6, 12). Many of them tend to form β sheet conformations that are prone to self-aggregate into different dimers, trimers, and tetramers, higher-order oligomers, protofibrils, and ultimately, typical 8-nm amyloid fibrils (7). There is clear evidence that the precise composition of these aggregates has a strong impact on their neurotoxicity (7, 13) and that different Aβ variants can trigger varying amounts of detrimental pro-inflammatory activities in astrocytes and microglia (14, 15). Their biochemical properties can vary significantly depending on the microenvironment in which they are generated, their amino acid composition, and their carboxyl terminus (13, 16). This makes the precise assessment of the Aβ structure for the etiology of AD extremely difficult.

Microglia are a key factor for AD. In a healthy brain, they provide tropic support to neurons while simultaneously surveying the central nervous system for pathological stimuli (14, 17). Upon activation, they undergo morphological changes and assume a reactive phenotype where they cease their supportive role (14). Instead, they obtain phagocytic and inflammatory functions, start to produce pro-inflammatory cytokines and chemokines such as IL-1β, IL-6, IL-8, and TNFα, and generate oxidative stress through the release of nitric oxide and reactive oxygen species (14, 18). Small oligomeric variants of Aβ are potent activators of microglia that lead to more severe neurotoxic outcomes than larger variants (19, 20). It is therefore crucial to understand how different Aβ variants interact with microglia to produce these neurotoxic effects. Unfortunately, interactions between Aβ and microglial cells are highly complex because Aβ acts through multiple pathways including TREM2 (Triggering receptor expressed on myeloid cells 2), TRPM2 (Transient receptor potential cation channel, subfamily M, member 2) (21), scavenger receptors such as CD36, MARCO (Macrophage receptor with collagenous structure), and RAGE (receptor for advanced glycation endproducts) (22), and pattern recognition receptors (PRR) such as Toll-like receptors (23), NOD-like receptors, (24) and FPRs (formyl peptide receptors) (12).

The activation of PRRs by Aβ has become a recent focus in AD research because they have a special ability to potently trigger the reactive state (14, 25). Among these PRRs, FPRs are one of the promising targets for AD research (12). They belong to a small gene family of G protein–coupled receptors with three members in humans and seven in mice (26). FPRs are primarily expressed in the innate and adaptive immune system, where they contribute to the detection and elimination of bacterial pathogens (27, 28, 29, 30). However, they are also expressed in a number of cell types in the brain such as microglia, astrocytes, and some specific subsets of neurons (31, 32, 33, 34, 35). Several independent lines of evidence support a significant contribution of FPRs to the pathological progression of AD. First, FPRs are highly upregulated in reactive glial cells at the site of senile plaques in human AD patients (36). Second Aβ_1–42_ induced a decrease of cAMP and an induction of ERK phosphorylation in rat microglia and astrocytes, which was inhibited by the synthetic FPR antagonist WRW4 (35, 37). Third, FPR2-dependent recognition of Aβ_1-42_ led to the induction of oxidative stress, the release of pro-inflammatory cytokines, and chemotaxis of neutrophils and murine glial cells (36, 38, 39, 40, 41). Fourth, *in vitro* expression studies demonstrated that Aβ_1–42_ can induce calcium mobilization and ERK signaling in cell lines that were transfected with human and murine FPR2 (35, 38, 42). In addition, FPRs mediate the intracellular uptake of Aβ_1-42_ in primary murine glia and in *in vitro* FPR expression systems (37, 43, 44, 45). Last but not least, treatment with the FPR antagonist Boc2 significantly ameliorated typical symptoms of AD such as cognitive impairment, decreased neuronal density, and Aβ plaque accumulation in an AD mouse model (46).

Despite these promising results, several key aspects of the interactions between Aβ and the different FPR variants are still insufficiently understood. For example, most studies on the interaction of Aβ with FPRs so far nearly exclusively focused on the role of FPR2. However, the human (h) gene family comprises the three members, hFPR1, hFPR2, and hFPR3. Thus, the potential of the other variants to interact with Aβ has not been extensively examined. Next, the murine (m) gene family comprises seven family members: mFpr1, mFpr2 mFpr3, mFpr-rs3, mFpr-rs4, mFrp-rs6, mFpr-rs7 (47). The only mouse gene that has a clear genetic ortholog inside the human gene family is mFpr1, which has a common ancestor with hFPR1 (48, 49). All others are paralogs, which evolved independently after the split of the human and mouse species (47), which makes it questionable to which extent data from mouse models can be transferred to humans. Finally, the interactions of FPRs with Aβ were nearly exclusively studied for Aβ_1-42,_ while data on the responses to other naturally occurring Aβ variants are lacking.

To address these questions, we used *in vitro* expression of human and mouse FPRs in HEK293T cells in combination with high-throughput measurements of intracellular calcium mobilization to systematically investigate the interactions between different Aβ variants with all human receptors and to compare their response with those of the relevant mouse receptors. In summary, these data provide the first clear evidence for a contribution of FPR1 and FPR3 to Aβ detection and identify N-abridged Aβ fragments as a previously unknown potent group of activators for FPRs. Our results reveal that in addition to mFpr2 and hFPR2, also mFpr1, hFPR1, and hFPR3 are capable to interact with Aβ_1-42._ Next, we can show that the solvent composition and manufacturer of the peptides critically influence the activation of FPRs by Aβ_1-42_. These variations likely depend on a different structural composition of Aβ from different sources. Furthermore, we demonstrate that human and mouse FPRs are also activated by Aβ_1-40_ and N-terminally abridged variants such as Aβ_11-40_ and Aβ_17-40_. Noteworthy, FPR1 is able to detect these peptides with up to 30-times higher sensitivity than Aβ_1-42_ and that these peptides induce calcium flux and chemotaxis in glial U87 cells that is likely mediated by hFPR1.

## Results

### Activation of formyl peptide receptors by Aβ is not restricted to FPR2

**Figure 1:**
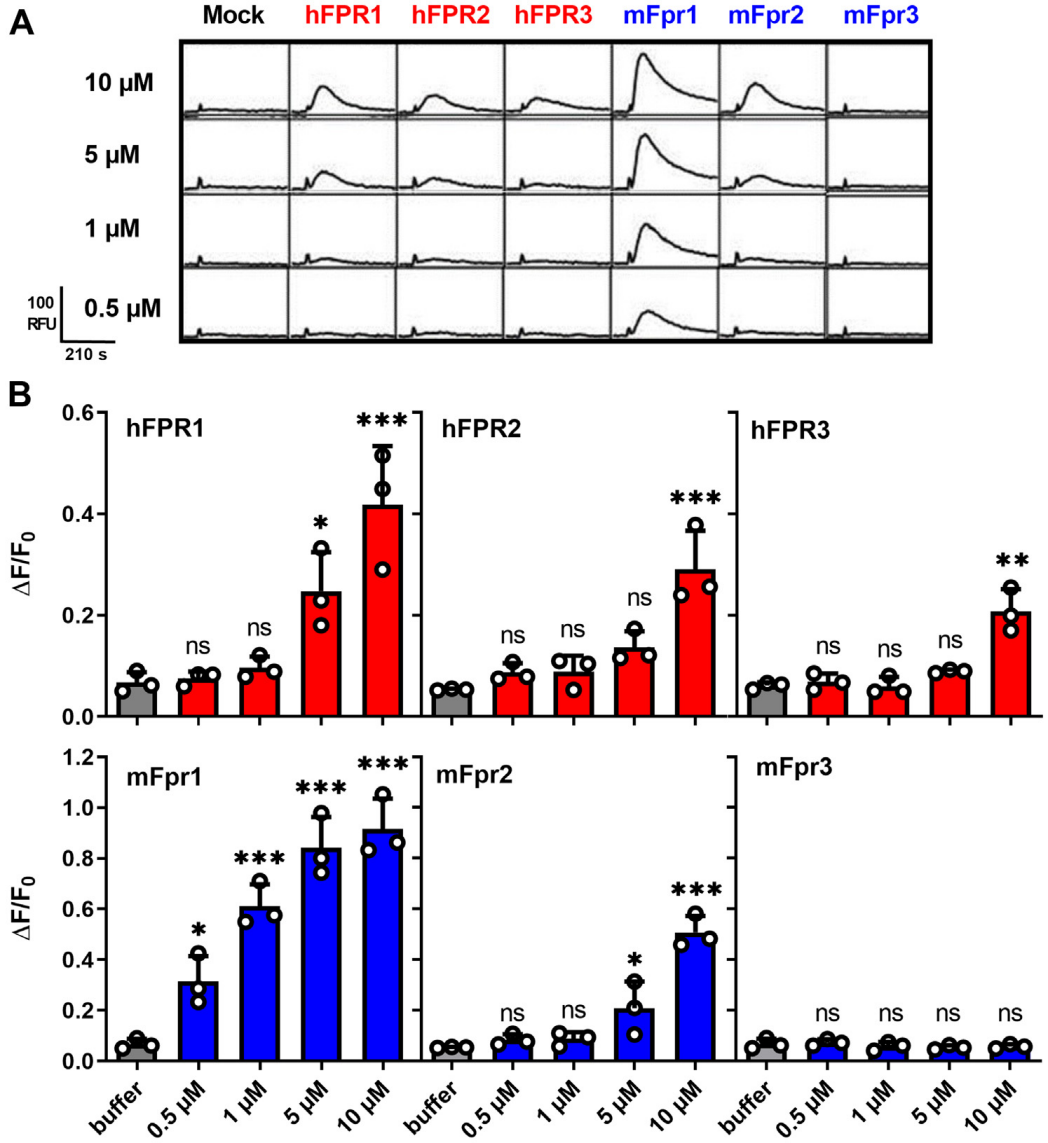
**Aβ**_**1-42**_**activates all human FPRs.***A*, representative Ca^2+^ traces of HEK293T cells transiently transfected with FPR plasmids or an empty vector (mock) after stimulation with Aβ_1-42_ obtained from P&E. *B*, mean peak Ca^2+^ responses of human (*red*) and mouse (*blue*) FPRs upon stimulation with different concentrations of Aβ_1-42_. Buffer (*gray*) denotes responses to the assay buffer without Aβ_1-42_. Bars represent mean values of three independent experiments (n = 3) carried out as technical duplicates (N = 2). All Error bars, S.D.; One-way ANOVA test, Dunnett post hoc test; ∗*p* ≤ 0.05; ∗∗*p* ≤ 0.01; ∗∗∗*p* ≤ 0.001; ns, no significance. Aβ, amyloid beta; FPR, Formyl peptide receptor.

We first investigated the ability of the human receptors hFPR1, hFPR2, and hFPR3 and their mouse counterparts’ mFpr1, mFpr2, and mFpr3 to detect Aβ. The further four existing mouse receptors mFpr-rs3, mFpr-rs4, mFrp-rs6, mFpr-rs7 were excluded from the analysis because they are not expressed in the brain (48) and likely do not mediate classical immune functions (50) but are responsible for the detection of yet largely unknown olfactory cues (50, 51). We first monitored changes in intracellular calcium levels of HEK293T cells transiently transfected with one of the different FPRs after application of Aβ_1-42_. Consistent with previous reports (36, 38), we observed that Aβ_1-42_ induced Ca^2+^ flux at low micromolar concentrations through mouse and human FPR2 (Fig. 1*A*). Surprisingly, we also observed a similar or even higher amount of activation of mFpr1, hFPR1, and hFPR3, whereas mFpr3 and mock-transfected negative controls did not respond. These data suggest that in addition to hFPR2, all other human FPRs are also able to respond to Aβ_1-42_. Noteworthy, subsequent concentration response tests showed that mouse and human FPR1 were even more sensitive and had a higher signal amplitude than any other receptor (Fig. 1*B*). Next, we also noticed some important differences between murine and human FPRs. First, we found that mFpr1 responded with an approximately tenfold higher sensitivity to Aβ_1-42_ than mFPR2, whereas the responses of human FPR1 were just slightly better than that the ones of hFPR2. This raises the possibility that mFpr1 might be more relevant for the physiological responses to Aβ_1-42_ in mouse models than its human ortholog. Second, we found an activation of human FPR3 by Aβ1_-42_ but did not detect a corresponding signal of its mouse counterpart. In summary, the observed species-dependent variations argue for a diverging importance of the different FPR paralogs for Aβ detection and signal transduction in between humans and mice, which should be further examined.

### Solvent and supplier variations can critically influence FPR responses to Aβ

**Figure 2:**
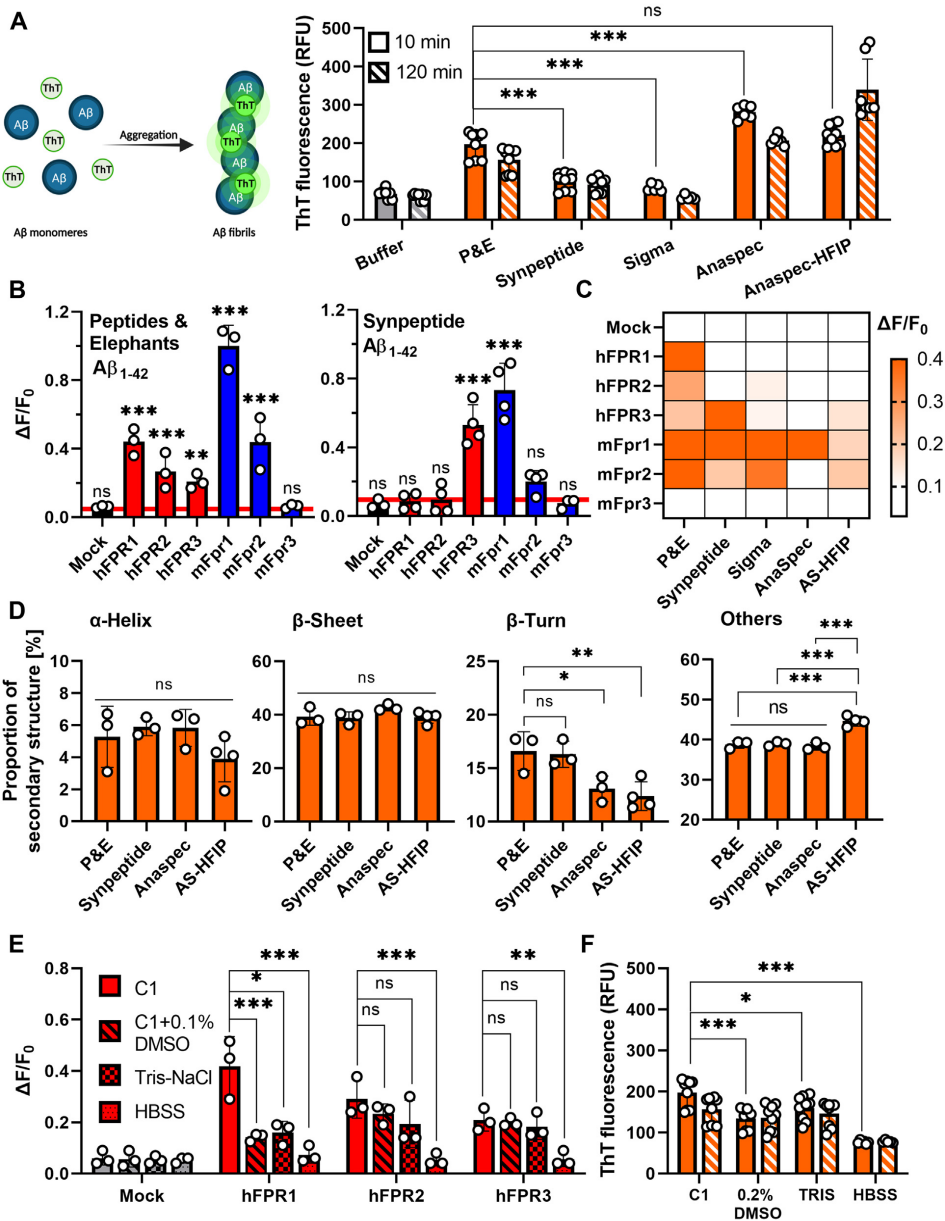
**Manufacturer- and solvent-effects on FPR activation by Aβ**_**1-42**_**.***A*, left: Schematic depiction of ThT aggregation assay. Right: Mean fluorescence of 22.5 μM Aβ_1-42_ in C1 buffers from different manufacturers in a ThT aggregation assay during the first 10 min (*clear bars*) versus fluorescence after 120 min (*striped bars*). Buffer refers to ThT fluorescence without addition of peptides (*gray bars*). All n = 3, N = 3, except for Sigma and Anaspec with n = 2, N = 3; One-way ANOVA test, Dunnett post hoc test. *B*, mean Ca^2+^ peak responses of human (*red*) or mouse (*blue*) FPRs to 10 μM of Aβ_1-42_ peptides obtained from Peptides & Elephants (P&E) and Synpeptide; n = 3, N = 2, One-way ANOVA test, Dunnett post hoc test in comparison to respective buffer controls. *C*, heat map of mean Ca^2+^ responses of FPRs elicited by Aβ_1-42_ peptides obtained from five different manufacturers. The scale ranges from *white* (no response) to *deep orange* (ΔF/F0 ≥ 0.4). Responses are shown in Figure S1B. *D*, secondary structure composition of four Aβ_1-42_ peptides analyzed by circular dichroism (CD) spectroscopy; n = 3, N = 1; One-way ANOVA test, Tukey post hoc test. *E*, mean Ca^2+^ peak responses of cells transfected with human FPRs (*red*) or mock (*gray*) towards 5 μM Aβ_1-42_ dissolved in the respective buffers; n = 3, N = 1, One-way ANOVA test, Dunnett post hoc test. *F*, comparison of ThT fluorescence of Aβ_1-42_ (P&E) dissolved in either C1, dimethyl sulfoxide (DMSO), Tris–NaCl, or HBSS. All assays were performed in the respective buffers. For experiments with peptides predissolved in DMSO, assays were conducted in C1 with a final concentration of DMSO: 0.2% (V/V). One-way ANOVA test, Dunnett post hoc test. All Error bars, S.D.; ∗*p* ≤ 0.05; ∗∗*p* ≤ 0.01; ∗∗∗*p* ≤ 0.001; ns, no significance. Aβ, amyloid beta; FPR, Formyl peptide receptor; ThT, thioflavin T.

**Supplemental Figure S1:**
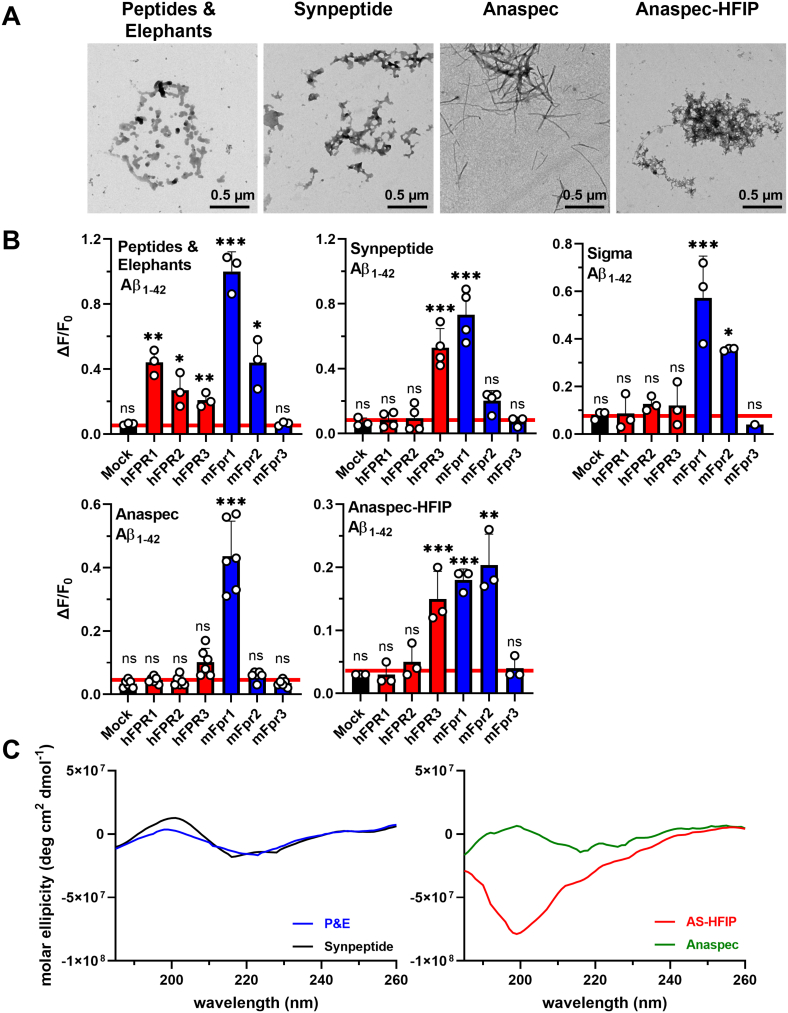
Manufacturer-dependent FPR activation by Aβ1-42 A, representative TEM images of Aβ1-42 obtained from either Peptides&Elepehants (P&E), Synpeptide, or Anaspec show different types of aggregates. AS-HFIP refers to a commercially available HFIP-treated peptide from Anaspec. Scale bars indicate 0.5 μm. B, mean Ca2+ peak responses of FPR-transfected HEK293T cells after stimulation with 10 μM Aβ1-42 obtained from different manufacturers. Colored bars indicate the response of human (red) or mouse (blue) FPRs, n=3, N=2. Data correspond to the heat map shown in Fig 2B. Bar graphs for P&E and Synpeptide are shown again for the sake of comparability. C, complete CD spectra of all four Aβ1-42 displayed in figure 2. N=3. All Error bars, S.D.; One-way ANOVA test, Dunnett post hoc test; ∗, p ≤ 0.05; ∗∗, p ≤ 0.01; ∗∗∗, p ≤ 0.001; ns, no significance

**Supplemental Figure S2:**
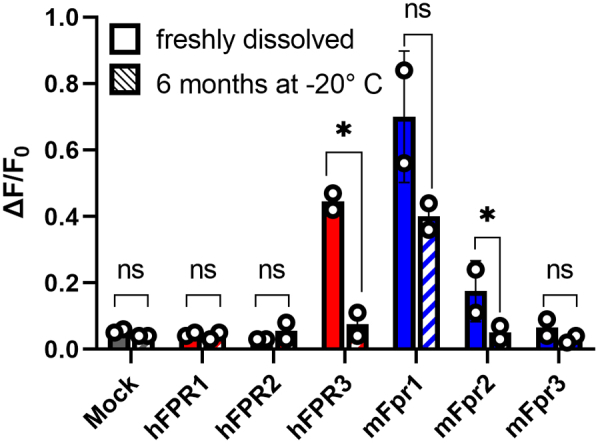
Alterations of FPR responses to Aβ1-42 after extended storage. Comparison of the Ca2+ responses of human (red) or mouse (blue) FPRs to 10 μM Aβ1-42 (Synpeptide) that were either freshly dissolved in the assay buffer C1 (clear bars) or had been stored in C1 at -20° C for 6 months without freeze-thaw cycles (striped bars), n=1, N=2. All Error bars, S.D.; ∗, p ≤ 0.05; ns, no significance.

**Supplemental Figure S3:**
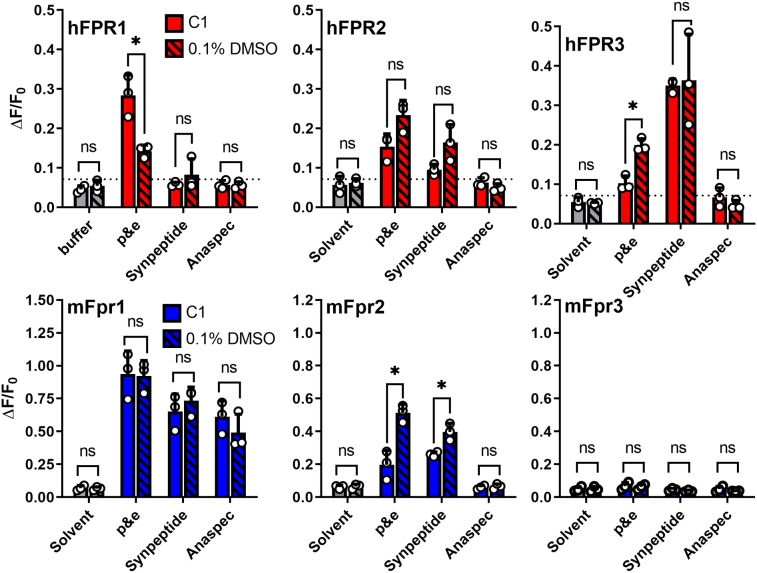
DMSO influences FPR activation by Aβ1-42. Mean Ca2+ peak responses of human (red) or mouse (blue) FPRs to 5 μM of Aβ1-42 peptides obtained from P&E, Synpeptide or Anaspec dissolved in either DMSO (striped bars) or C1 (clear bars), n=3, N=2. All Error bars, S.D.; ∗, p ≤ 0.05; ns, no significance.

A detailed comparison of our results with other publications revealed some inconsistencies in the current literature. While our observation that Aβ_1-42_ induces Ca^2+^ flux through human and mouse FPR2 in micromolar concentrations is in accordance with all previous studies (38, 42), our study is the first to report of an activation of hFPR3. Next, our notion that mouse and human FPR1 are activated by Aβ_1-42_ is in line with the results reported by Le *et al.* and Slowik *et al*. (35, 42). However, in these studies, the hFPR1 responses were far less pronounced and a study by Tiffany *et al.* even failed to detect any activation of mouse and human FPR1. A careful comparison of the methods used in these and other reports for possible explanations revealed three major potential sources. First, researchers frequently use dimethyl sulfoxide (DMSO) to dissolve Aβ_1-42_, which can be critical in the case of FPRs because DMSO is an agonist for FPR1 and FPR2 and may therefore affect the Aβ_1-42_-evoked signals (50). Second, varying types of physiological assay buffers and solvents were used, which might be critical in the case of Aβ_1-42_ because this peptide tends to form diverse kinds of aggregates in different assay buffers (20, 52, 53). Third, Aβ_1-42_ was obtained from different sources, which raises the possibility that variations in the production processes may have influenced the peptide aggregation status. We therefore decided to carefully investigate the influence of these factors on the FPR responses. To examine the impact of peptide synthesis on the receptor responses, we ordered four additional Aβ_1-42_ peptides from three additional sources (Anaspec, Sigma-Aldrich, and Synpeptide) and compared them to the peptide that we obtained from our initial supplier Peptides&Elephants (P&E). A second peptide from Anaspec that was Hexafluoroisopropanol (HFIP) treated was used as positive control for aggregation because this peptide is known to readily form aggregates. Using a thioflavin T (ThT) assay (Fig. 2*A*), we first compared the aggregation status 10 min after dissolving the peptides with their kinetics over the course of the next 2 hours because we estimated that this would be the maximal time span to perform our calcium imaging experiments. We already observed clear differences between the four supposedly identical peptides in the 10 min after dissolving them in our assay buffer. In comparison to the peptide from P&E, Aβ_1-42_ from Synpeptide and Sigma-Aldrich showed an approximately 30% to 50% lower aggregate content, whereas the non-HFIP–treated peptide from Anaspec was approximately 30% more pre-aggregated. The kinetics in the first 2 h revealed that the aggregation control with the HFIP-treated peptide showed the expected increase, whereas the status of Aβ_1-42_ from Sigma-Aldrich and Synpeptide did not drastically change. The non-HFIP–treated Anaspec peptide even showed a reduction of fluorescence. In transmission electron microscopy (TEM, Fig. S1*A*), Aβ_1-42_ from P&E, Synpeptide, and the HFIP-treated Anaspec peptide displayed a somewhat similar amorphous morphology, while the non-HFIP–treated Anaspec peptide seemed to directly form long fibers, which may explain the high starting fluorescence in our ThT assay. Together, these results indicate a considerable amount of heterogeneity in the secondary and tertiary structure of peptides from different sources. In line with this, Ca^2+^ flux measurements also revealed clear differences in the response pattern of mouse and human FPRs to these peptides (Figs 2, *B* and *C* and S1*B*). Aβ_1-42_ from Sigma-Aldrich elicited a similar response pattern as that of P&E. However, it failed to activate hFPR1. When using Aβ_1-42_ from Synpeptide, the responses of hFPR1 and hFPR2 were lost. The non-HFIP–treated peptide from Anaspec potently activated mFpr1 but failed to activate any other mouse or human FPR, whereas the HFIP-treated version weakly activated hFPR3, mFpr1, and mFpr2. Thus, variations in the manufacturing process are critical factors that are capable to strongly affect the activation pattern of FPRs. To evaluate the secondary structure of our peptides, we next performed CD spectroscopy on Aβ_1-42_ peptides from P&E, Synpeptide, and the two variants from Anaspec, all dissolved in the assay buffer C1 (Figs. 2*D* and S1*C*). All tested peptides showed similar proportions of α-helix and β-sheet but differed in the amount of β-turns and unstructured sequences. In comparison with the two different Anaspec peptides that elicited only relatively weak calcium responses, the Aβ_1-42_ peptides from P&E and Synpeptide, which were able to more robustly activate FPRs, displayed significantly elevated β-turn proportions. This suggests that increased proportions of β-turn conformation in Aβ peptides may improve their interaction with FPRs. We next examined the influence of different assay buffers on FPR signals. To this end, we first compared the FPR responses to Aβ_1-42_ from P&E dissolved in our assay buffer C1 with those of the two frequently used buffers Hank's balanced salt solution (HBSS) and Tris–NaCl (Fig. 2*E*). We detected the most robust activation of human and mouse FPRs in C1. The use of Tris–NaCl lead to an approximately 50% and 20% reduction of the signal amplitude of hFPR1- and hFPR2-based response, respectively but did not significantly alter the responses of hFPR3. Use of HBSS, by contrast, resulted in a complete loss of nearly all FPR responses. Interestingly, this was clearly correlated with a strong reduction in the capability to form aggregates (Fig. 2*F*). Furthermore, we even observed that a longer storage of the dissolved peptides in the freezer can affect the response pattern (Fig. S2). Next, we carefully examined the effect of the commonly used solvent DMSO on the Aβ_1-42_ response pattern of the individual FPRs (Fig. S3). Again, we observed a number of subtle changes in the FPR response patterns that were difficult to predict because they largely depended on a specific combination of receptor subtype and peptide source. For example, DMSO diminished the hFPR1 response to Aβ_1-42_ from P&E by 40%, whereas it increased the response of hFPR3 by 32%. Despite these variations, our data clearly demonstrate that mFpr1 and hFPR3 were activated under almost all conditions. However, the overall activation pattern of FPRs showed considerable alterations that depend on manufacturing source, assay buffer conditions, storage time, and the pretreatment of Aβ_1-42_ with different solvents. Thus, there is a clear need for standardization and careful description of all Experimental procedures.

### N-abridged Aβ-fragments are potent activators of mouse and human FPR1

**Figure 3:**
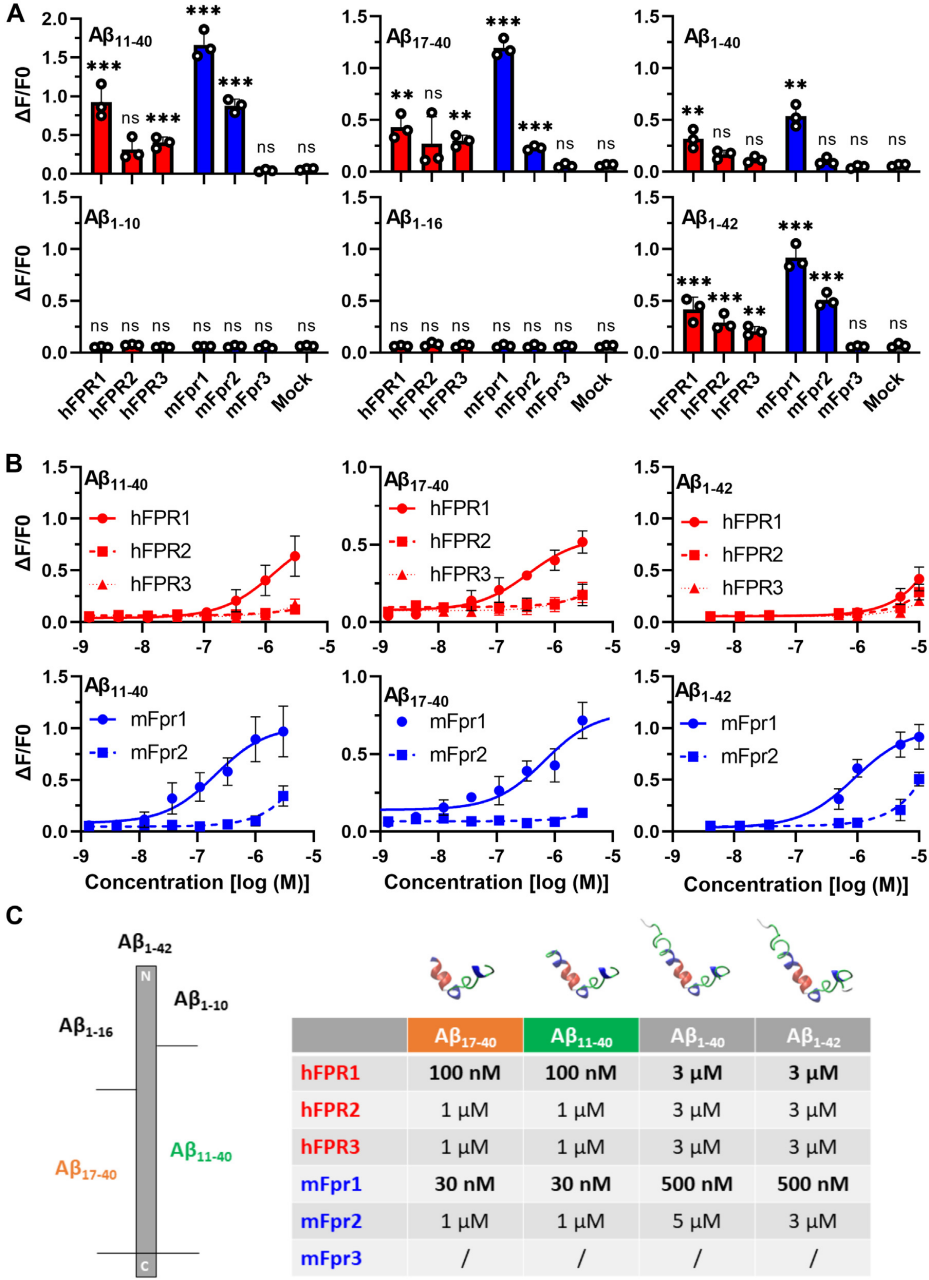
**Naturally occurring N-abridged Aβ fragments activate FPR1 tenfold better than Aβ**_**1-42**_**.***A*, comparison of mean Ca^2+^ peak responses of human (*red*) or mouse (*blue*) FPRs to a stimulation with 10 μM Aβ_1-40_ and Aβ_1-42_ or 5 μM of the natural occurring N-abridged variants Aβ_11-40_ and Aβ_17-40_ or with 10 μM of the C-abridged variants Aβ_1-10_ and Aβ_1-16_. *Colored bars* indicate responses of human (*red*) or mouse (*blue*) FPRs, n = 3, N = 1. *B*, concentration response curves of selected variants, n = 3, N = 1. *C*, *left*: scheme indicating the size and location of the different Aβ variants. *Right:* Table depicting the proposed 3D-structures and thresholds for minimal detectable activation during Ca^2+^ imaging of the responding Aβ variants. All Error bars, S.D. One-way ANOVA test, Dunnett post hoc test; ∗∗*p* ≤ 0.01; ∗∗∗*p* ≤ 0.001; ns, no significance. Aβ, amyloid beta; FPR, Formyl peptide receptor.

**Supplemental Figure S4:**
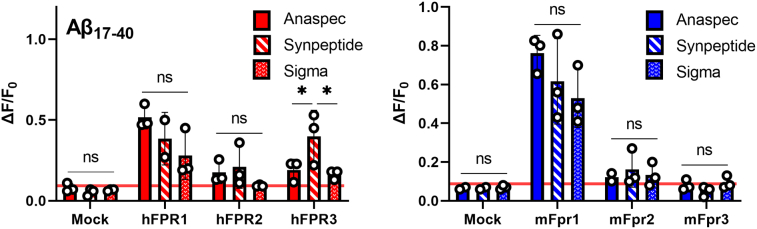
Aβ17-40 is less susceptible to manufacturer-dependent effects than full length Aβ. Mean Ca2+ peak responses of FPR-transfected HEK293T cells to 5 μM of Aβ17-40 from three different manufacturers. Clear bars, Aβ17-40 from Anaspec. Striped bars, Aβ17-40 from Synpeptide. Dotted bars: Aβ17-40 from Sigma-Aldrich, n=3, N=1. All Error bars, S.D.; One-way ANOVA test, Dunnett post hoc test; ∗, p ≤ 0.05; ns, no significance.

Under *in vivo* conditions, not only Aβ_1-42_ but several other fragments such as Aβ_1-40_, Aβ_11-40_, and Aβ_17-40_ are also generated during the cleavage of APP and its subsequent processing (12, 54, 55). Thus, it is conceivable that FPRs can also interact with some of these fragments. To test this hypothesis, we investigated the response of FPRs to the commonly found Aβ_1-40_ the N-abridged variants Aβ_11-40_ and Aβ_17-40_ and the C-terminally abridged fragments Aβ_1-10_ and Aβ_1-16_ (Fig. 3*A*). We observed that longer C-terminal deletions tend to be detrimental for the activation of FPRs because fragments such as Aβ_1-10_ and Aβ_1-16_ failed to induce any Ca^2+^ mobilization. In sharp contrast, the two tested N-abridged variants Aβ_11-40_ and Aβ_17-40_ elicited responses on all FPRs that we could previously activate with the full-length Aβ_1-42._ Unlike the responses to the full-length Aβ_1-42_, the FPR responses to these shorter fragments seemed to be less prone to manufacturer-dependent variations (Fig. S4). Surprisingly, N-terminal deletions tended to strongly improve the interaction with FPR1. Concentration response curves revealed that mouse and human FPR1 could detect both N-abridged fragments with more than 10-fold higher sensitivity than the full-length peptide Aβ_1-42_. (Fig. 3, *B* and *C*). Mouse and human FPR2 only responded to micromolar concentrations of these peptides (Fig. 3*B*). The observation that Aβ_11-40_ and Aβ_17-40_ can already induce an activation of mouse and human FPR1 at 30 to 100 nanomolar concentration raises the possibility that the detection of these short fragments through FPR1 might be even more relevant for the physiological response than the activation of FPR2 by longer Aβ at micromolar concentration.

### N-abridged Aβ variants are potent activators for a human glia cell line

**Figure 4:**
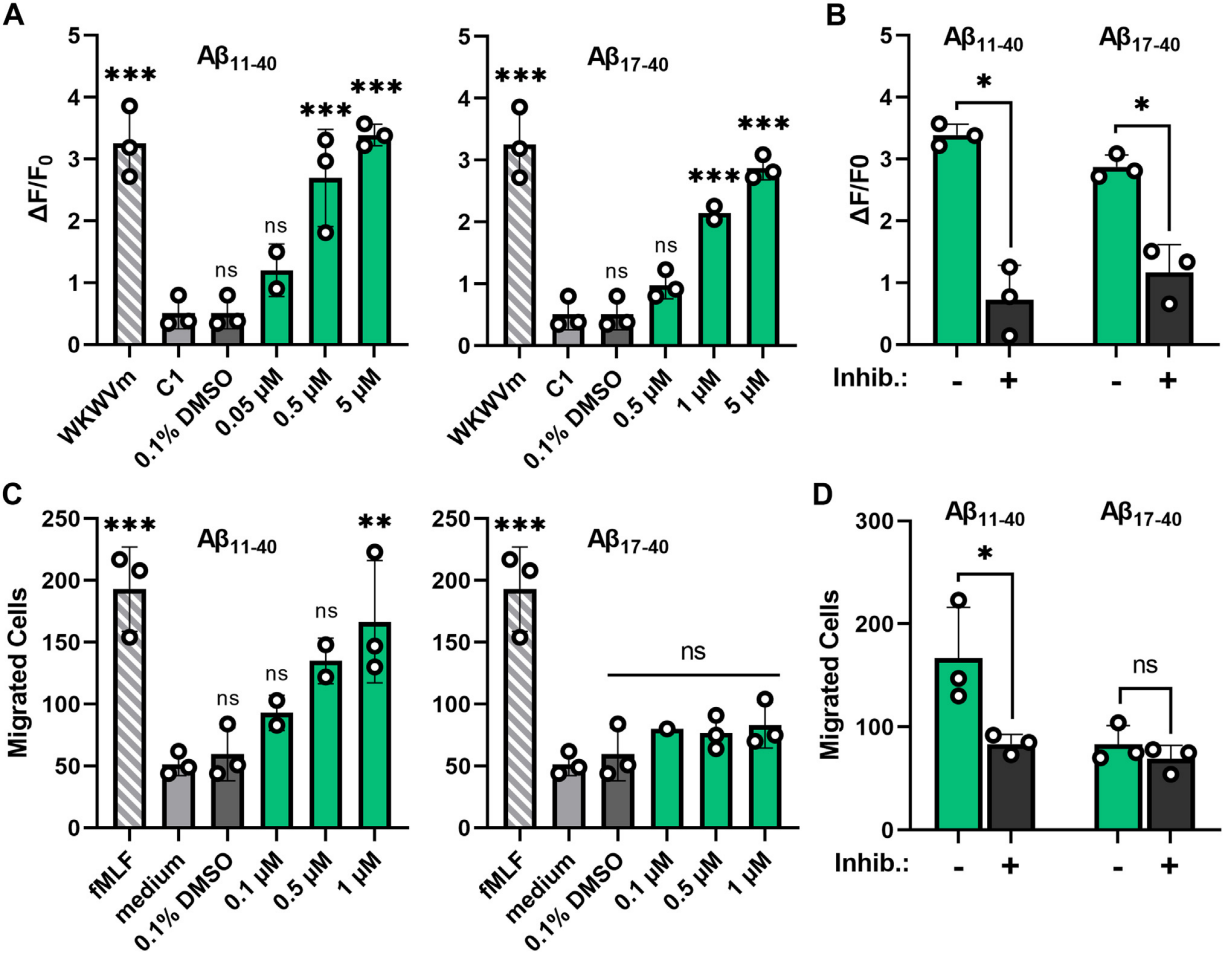
**N-abridged Aβ peptides induce hFPR1-dependent responses in glial U87 cells.***A*, mean Ca^2+^ peak responses of U87 cells after stimulation with different concentrations of Aβ_11-40_ or Aβ_17-40._*Striped bars* indicate the response towards 10 μM of the positive control WKWVm-NH_2_, *light gray* and *dark gray* bars indicate negative controls; n = 3, N = 1. *B*, comparison of the Ca^2+^ responses upon stimulation with either 5 μM Aβ_11-40_ or Aβ_17-40_ alone (*green bars*) or in the presence of 10 μM of the competitive FPR-antagonist tBoc2 (*black bars*); n = 3, N = 1. *C*, dose-dependent chemotaxis of U87 cells upon stimulation with either Aβ_11-40_ or Aβ_17-40_. Each *bar* represents the number of cells that migrated through a porous membrane towards the respective stimuli. *Green bars* indicate migration towards N-abridged fragments, *striped bars* display migration towards the positive control 1 μM fMLF, *light gray bars* show migration without stimuli, and *dark gray bars* represent the response to 0.1% DMSO, n = 3, N = 1. *D*, migration of U87 cells that were either untreated (*green bars*) or treated with 10 μM tBoc2 (*black bars*) towards 1 μM of Aβ_11-40_ or Aβ_17-40_; n = 3, N = 1. All Error bars, S.D. One-way ANOVA test, Dunnett post hoc test for A and C and *t* test for B and D; ∗*p* ≤ 0.05; ∗∗*p* ≤ 0.01; ∗∗∗*p* ≤ 0.001; ns, no significance. Aβ, amyloid beta; FPR, Formyl peptide receptor.

**Figure 5:**
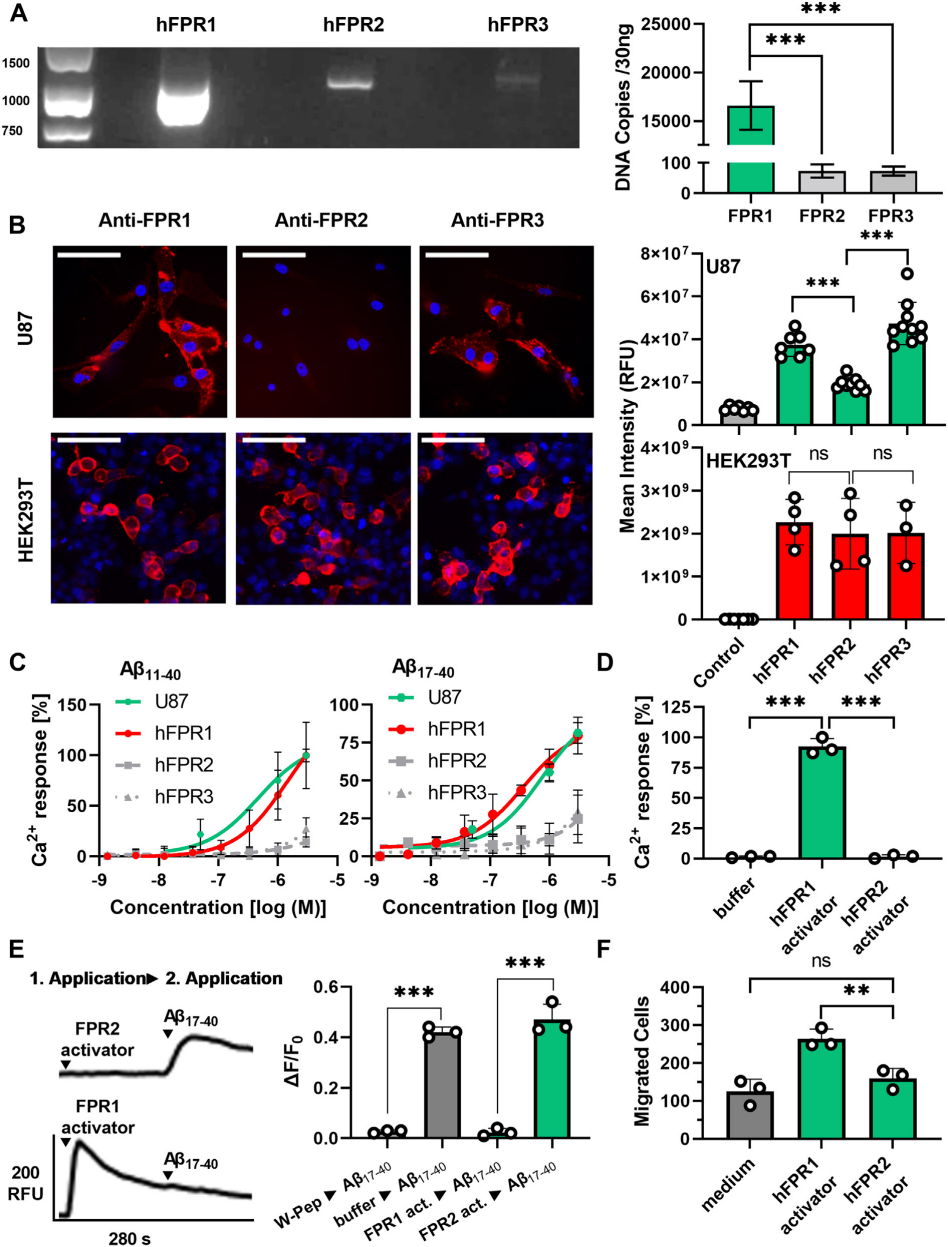
**The activation of U87 cells by N-abridged Aβ peptides depends on hFPR1.***A*, PCR experiments show that U87 cells contain high mRNA levels of hFPR1 but only low amounts of FPR2 and FPR3. Left: Representative image of gel electrophoresis after reverse transcription polymerase chain reaction with primers for all hFPRs. Right: Quantification of complementary DNA for all FPRs obtained through reverse transcription quantitative polymerase chain reaction, n = 5, N = 2. Details on the primer efficiency, specificity, and linearity are given in Figures S6 and S7. One-way ANOVA test, Dunnett post hoc test. *B*, *left*: representative immunocytochemistry staining of U87 cells and transfected HEK293T cells with FPR subtype–specific antibodies (*red*) and nuclei staining (*blue*). For visibility, brightness and contrast were adjusted for U87 cells and HEK293T cells differently; for absolute intensity comparison pictures with equal settings for exposure time, brightness and contrast pictures are shown in Figure S5. Scale bars indicate 100 μm. Right: Quantification of the mean FPR staining in U87 cells (*green*) in comparison to FPR-transfected HEK293T cells (*red*). Analysis was performed on images acquired with the same settings. n = 2, N = 5 for U87 cells and n = 2, N = 2 for HEK293T cells. Evidence for closely similar staining intensities of FPR1 and FPR2 by the used protocols is shown in Figure S5. One-way ANOVA test, Tukey post hoc test. *C*, the concentration-dependent Ca^2+^ responses of HEK293 cells and U87 cells towards the N-abridged fragments Aβ_11-40_ and Aβ_17-40_ reveal a clear correlation with the hFPR1 response, n = 3, N = 1. *D*, U87 cells respond to the potent hFPR1 activator SP6 (10 nM) but not to a potent hFPR2 agonist SP4 (10 nM) in calcium imaging experiments, n = 3, N = 3. One-way ANOVA test, Dunnett post hoc test. *E*, Cross-desensitization experiment of U87 cells show that the Ca^2+^ responses towards Aβ_17-40_ are abolished by the FPR1 activator SP6 but not by the hFPR2 agonist SP4. Representative Ca^+^ traces (*left*) and mean Ca^2+^ peak responses (*right*) to a secondary Aβ_17-40_ stimulus after pre-application of SP4 or SP6 as a first stimulus, n = 1, N = 3; *t* test. *F*, U87 migrate towards the hFPR1-stimulus SP6 (10 nM) but do not respond to the hFPR2 agonist SP4 (10 nM), n = 3, N = 1. One-way ANOVA test, Tukey post hoc test. All Error bars, S.D. ∗*p* ≤ 0.05, ∗∗*p* ≤ 0.01, ∗∗∗*p* ≤ 0.001; ns, no significance. Aβ, amyloid beta; FPR, Formyl peptide receptor.

**Supplemental Figure S5:**
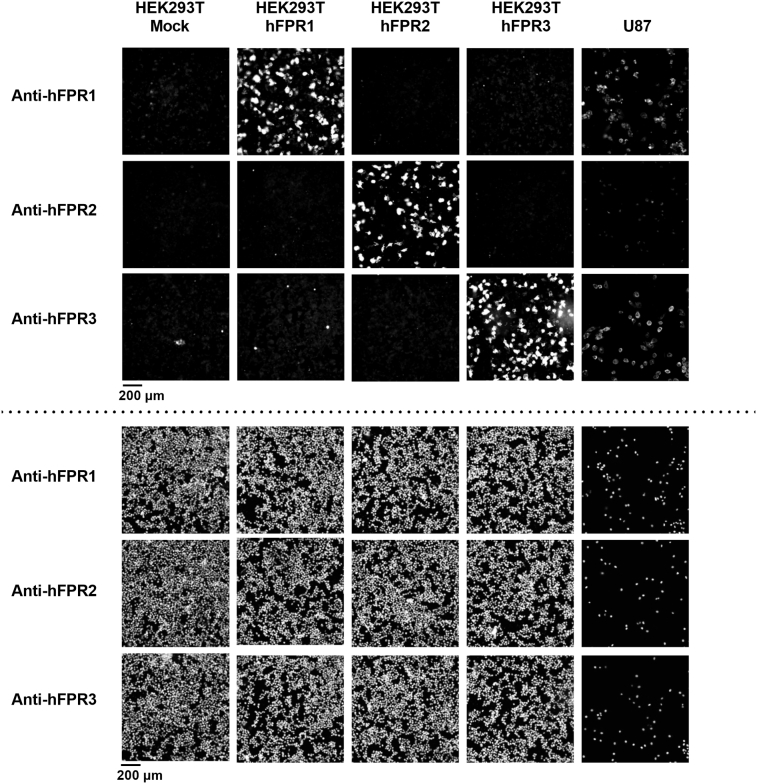
Validation of immunofluorescence staining with FPR subtype-specific antibodies. Top: Comparison of immunofluorescence staining intensity of FPR-transfected HEK293T and U87 cells with receptor subtype specific antibodies. The hFPR1 (1 μg/ml), hFPR2 (0.2 μg/ml) and hFPR3 (1 μg/ml) antibodies were used. Note that the absolute staining intensities of all hFPRs in HEK293T cells were highly similar, however a strong reduction of hFPR2 staining in U87 cells is observed. No cross reactivity was detected. All images were acquired with the same settings and are displayed with the same illumination, brightness and contrast. Evidence for similar cell surface expression and production rates for hFPR1 and hFPR2 can be found in supporting Fig. S8. Bottom: corresponding nuclei staining with Hoechst 33342 demonstrating a comparable amount of total cell number in all visual fields.

**Supplemental Figure S6:**
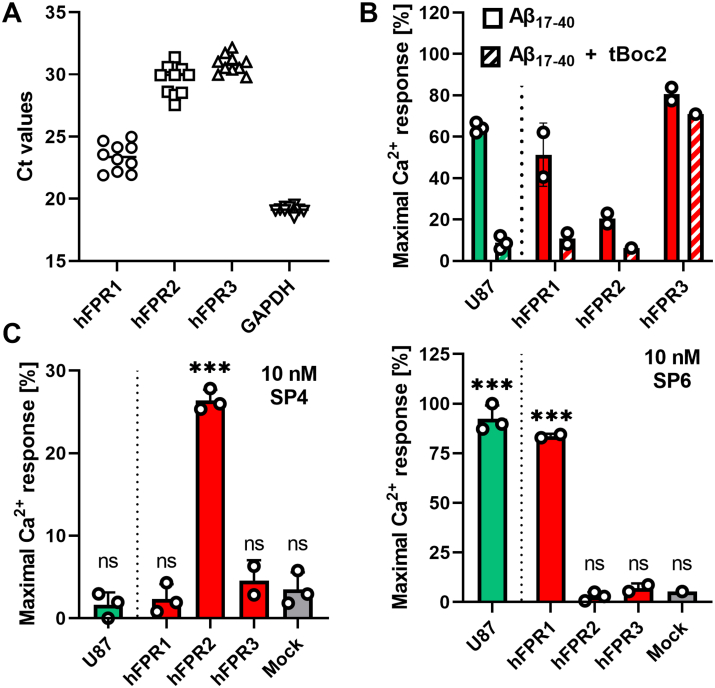
FPR1 is the dominant receptor in U87 cells. A, Ct values of all human FPRs from the RNA of U87 cells obtained through RT-qPCR, n=5, N=2. B, Ca2+ responses of U87 cells (green) or FPR-transfected HEK293T cells (red) towards 5 μM Aβ17-40 with (clear bars) or without (striped bars) co-application of 10 μM tBoc2. Signals were normalized to responses of the respective cells towards 10 μM WKYMVm, n=1, N=2 for HEK293T cells and N=3 for U87 cells. C, mean Ca2+ peak responses of U87 cells (green) or FPR-transfected HEK293T cells (red) that were treated with 10 nM SP4 (left) or 10 nM SP6 (right), n=1, N=3. All Error bars, S.D. One-way ANOVA test, Dunnett post hoc test; ∗, p ≤ 0.05, ∗∗, p ≤ 0.01, ∗∗∗, p ≤ 0.001; ns, no significance

**Supplemental Figure S7:**
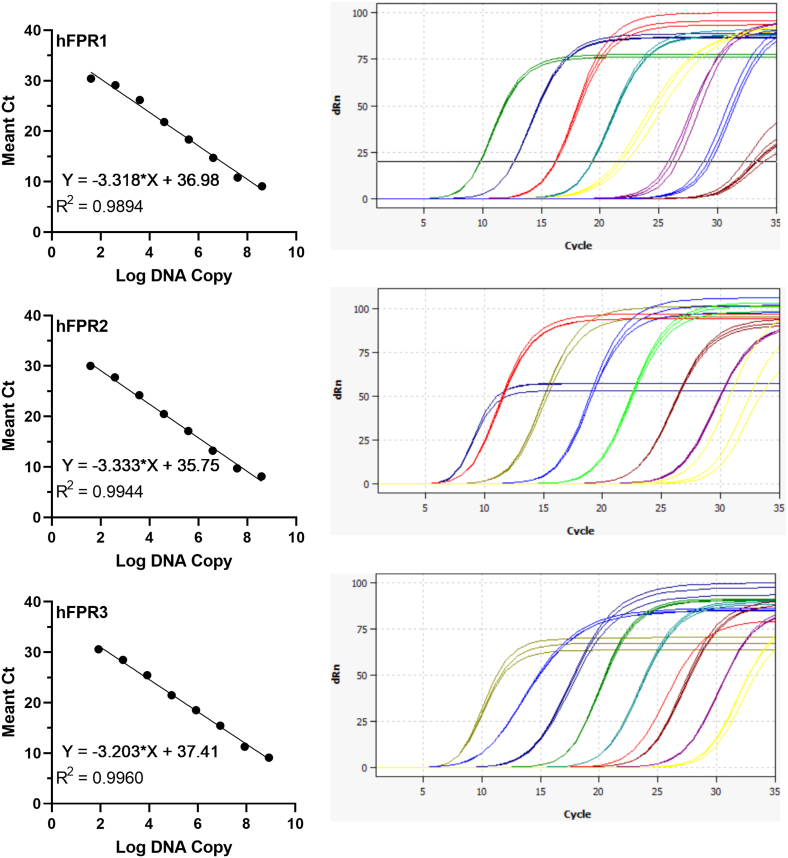
Validation of RT-qPCR primers for all human FPRs. Left: mean standard curves, regression line, and amplification efficiency (n =3, N=3) generated by using a 10-fold serial dilution of a target DNA template starting with 0,1 ng of a purified and sequenced PCR product in t-RNA. Ct values were plotted against the log of template quantity for each dilution. Representative PCR products for all primer sets were controlled by gel electrophoresis and sequencing for their specificity. Right: A representative data set of an amplification curve from these dilution series for each primer.

So far, our study focused on the characterization of the FPR pharmacology in an *in vitro* over-expression–based system. Despite the strength of this system to dissect responses of the individual FPRs to Aβ, we cannot exclude that differences in the signal transduction system of HEK293T cells or the overexpression of the receptors may affect our pharmacological results. We therefore sought to validate our results in a more biologically relevant setting. To this end, we examined the responses elicited by Aβ_11-40_ and Aβ_17-40_ in U87 cells (Fig. 4) that are commonly used as a human glial cell model and are known for their natural expression of FPRs (56, 57). We first used reverse transcription polymerase chain reaction and reverse transcription quantitative polymerase chain reaction to examine the expression of the individual FPRs in these cells. Our data revealed that these cells show high mRNA levels of hFPR1 but only a very modest expression of hFPR2 and hFPR3 (Figs. 5*A* and S6*A* and S7). Immunocytochemistry with receptor subtype-specific antibodies (Figs. 5*B* and S5) confirmed a robust expression of hFPR1 and lower amounts of hFPR2. Interestingly, hFPR3 showed a relatively abundant protein amount despite low amounts of mRNA which is consistent with similar findings for mFpr3 expression in mouse immune cells (58).

Calcium imaging experiments revealed that a stimulation with Aβ_11-40_ and Aβ_17-40_ resulted in a pronounced activation of U87 cells (Fig. 4, *A* and *B*) that were closely similar to the typical FPR time kinetics (50) observed in the transfected HEK cells (Fig. 1). In chemotaxis experiments, only Aβ_11-40_ but not Aβ_17-40_ induced migration of these cells (Fig. 4, *C* and *D*). Both, the calcium signals and the chemotaxis, were FPR dependent because a co-application of tBoc2, which is a competitive antagonist of FPR1 and FPR2 but does not inhibit FPR3 (Fig. S6*B*), completely abolished the responses. To address the question which FPR exactly is responsible for the observed results, we first compared the U87 concentration response curves of Aβ_11-40_ and Aβ_17-40_ with those of FPR-transfected HEK293T cells. We found them to be remarkably similar to hFPR1 (Fig. 5*C*). Next, we used receptor subtype-specific activators to test which receptor can trigger a calcium signal. To this end, we challenged U87 cells and hFPR-transfected HEK293T cells with two bacterial signal peptides SP6 and SP4 at concentrations where they would only activate either hFPR1 or hFPR2, respectively (50). Of note, both compounds would not activate hFPR3 (Fig. S6*C*). In line with our previous notion that responses to the N-abridged Aβ variants likely depend on hFPR1, the U87 cells only responded to the hFPR1-specific SP6 stimulus with a calcium signal but not to the hFPR2-specific SP4 stimulus (Figs. 5*D* and S6*C*). Next, we performed cross-desensitization experiments, which showed that responses of U87 cells towards Aβ_17-40_ were absent after pre-application of the FPR1-specific agonist SP6 but were not influenced by pre-application of the FPR2-specific agonist SP4 (Fig. 5*E*). Finally, in the chemotaxis assay, U87 cells only migrated towards the FPR1-specific SP6 but not towards the FPR2-specific SP4 (Fig. 5*F*). Taken together, these experiments strongly argue that the sensitive calcium and chemotaxis responses of U87 to N-abridged Aβ variants depend on an activation of hFPR1.

## Discussion

In summary, our study provides a systematic overview over the capability of different mouse and human FPRs to interact with Aβ in different buffer systems. To our knowledge, we are the first to report an activation of hFPR3 by Aβ and the first to identify N-abridged Aβ fragments as activators of FPRs. Moreover, our data show that hFPR1 and its mouse ortholog mFpr1 can recognize N-terminally abridged fragments such as Aβ_11-40_ and Aβ_17-40_ at drastically lower concentrations than longer peptides such as Aβ_1-40_ and Aβ_1-42_, which raises the possibility that FPR1 might be more relevant for the pro-inflammatory physiological responses to Aβ than FPR2. Interestingly, several independent lines of evidence from literature are highly consistent with this hypothesis. First, FPR1 is already expressed at the resting state of glial cells and is further upregulated during their transformation into the reactive state (59). Next, mFpr1 is highly upregulated in the cortex and hippocampus of transgenic APP/PS1 mice, which are a commonly used AD animal model (35). Moreover, FPR1 has been shown to mediate typical pro-inflammatory effects in glial cells that are also commonly observed in AD such as generation of oxidative stress, release of inflammatory cytokines and chemokines, and the induction of cell migration (14, 35), whereas several reports suggest an anti-inflammatory role of FPR2 (41, 44, 60). Next, treatment of APP/PS1 mice with the competitive FPR antagonist tBoc2 that preferentially binds to FPR1 (26) leads to reduced microglia reactivity, decreased neuronal pathology, and improved cognitive performance (46). Taken together, these findings strongly support our notion that FPR1 and other FPR variants contribute to neuroinflammation of AD through their ability to sense different Aβ variants. Of note, this contribution might be rather complex because we here observed that abridged variants sometimes trigger partially different signaling pathways (*e.g.*, calcium signaling only for Aβ_17-40_ or calcium signaling and migration for Aβ_11-40_) likely through exclusive activation of the identical receptor. Next, a given Aβ peptide will show a concentration-dependent interaction with different FPR receptors. Finally, other pro- and anti-inflammatory FPR ligands such as Annexin A1 (44, 61), Lipoxin A 4 (62, 63), Resolvin (63), or mitochondrial peptides (64) may further modulate these signals depending on their local concentration. However, the observed 10- to 30-fold higher affinity of FPR1 to N-abridged fragments insinuates that such fragments might be of high pathological relevance for AD if they occur under physiological conditions in sufficient concentrations. Indeed, several studies suggest that Aβ_11-40/42_ is present in the cerebrospinal fluid at similar concentrations as Aβ_1-42_ and is furthermore an integral part of senile plaques (54, 65, 66). Aβ_17-40/42_ is thought to occur at 4-fold higher rates than Aβ_1-42_ and Aβ_11-40/42_, however, its exact quantification is difficult and therefore often disregarded (55). In accordance with our data, both Aβ_11-40_ and Aβ_17-40_ were shown to activate glial cells, which is associated with the neurotoxic effects seen in AD (53, 67). Moreover, our observation that Aβ_11-40_– but not Aβ_17-40_–induced chemotaxis of U87 cells is in line with previous results that show that Aβ_17-40/42_ and Aβ_17-43_ only lead to a partial activation of human glial cells (53, 68). Thus, to the best of our knowledge, our data are consistent with a model in which recognition of these N-abridged Aβ variants *via* FPR1 could thus occur under physiological conditions.

**Figure 6:**
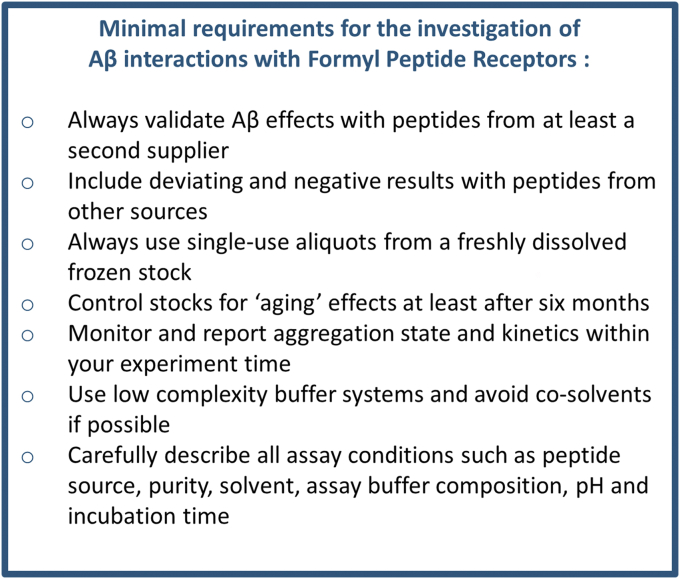
**Minimal requirements for the investigation of Aβ interactions with FPRs.** Aβ, amyloid beta; FPR, Formyl peptide receptor.

Finally, our results clearly demonstrate that the precise FPR responses towards different Aβ variants are highly subjectable to solvent- and manufacturer-dependent effects. Factors such as the secondary structure, 3D conformation, and aggregation therefore seem to be of high importance for the precise activation pattern, and different receptor subtypes seem to prefer different peptide structures or exposed surfaces. Multiple previous studies have already shown that different buffer systems and solvents can critically influence properties of Aβ such as its aggregation kinetics and the structure of its aggregates (20, 52, 53). For example, Szczepanik *et al.* (53) observed differences in Aβ-dependent cytokine release depending on the solvent of their peptides. We assume that variations within our measurements occur due to formation of aggregates and fibers captured by TEM images and variations in the secondary structure as indicated by our CD. In accordance with this idea, in previous studies, both monocytes and microglia lost FPR-dependent signaling with progressing aggregation of Aβ_1-42_ (42, 69). Of note, it is conceivable that chemical modifications of the peptide, for example, oxidation of methionine at position 35, racemization, or isomerization may contribute to the functional differences that we observed for an identical peptide in different buffer systems. This needs to be further examined. However, our data also show that it is necessary to develop a generally accepted standardized protocol for the investigation of FPR-related effects on Aβ peptides. In many fields, the use of similar assay conditions has thus improved the reproducibility and quality of data (20). Based on our current results, we would like to recommend the following protocol (see Fig. 6). We propose to validate any observed Aβ effects with a peptide from second supplier. Next, we would like to encourage researcher to also include negative results with peptides from other sources. All experiments should be performed using single use aliquots from a freshly dissolved frozen stock. Next, any stocks that are stored should be examined for “aging” effects. We also suggest the use of low complexity buffer systems such as our C1 buffer and avoid cosolvents such as DMSO if possible. In case DMSO or other cosolvents are used, their effects on aggregation and physiological response need to be controlled and reported. All experiments should be performed within the same standardized time in order to have a similar amount of aggregation. Finally, we suggest to always include at least some data on the aggregation status and time kinetics of the peptides in a given buffer system, which can be easily done with simple inexpensive methods such as ThT assays. Moreover, we would like encourage a very detailed description of all experimental conditions that may affect the aggregation as Supporting material. Especially, the peptide source including lot numbers, purity, used solvents, precise assay buffer composition, pH, incubation time, storage time, storage conditions, and final concentration of cosolvents in an assay should be reported because this all can critically influence the results.

## Experimental procedures

### Cloning of human and murine FPR genes

Human hFPR1, hFPR2, and hFPR3 and murine mFpr1, mFpr2, and mFpr3 were amplified from genomic human or C57BL/6J murine DNA, respectively and subcloned into pcDNA3.1^(+)^ (Invitrogen) as previously described (50).

### Ligands and chemicals

Purity of all peptides was at least >95%. Details on manufacturer, Catalog numbers, lot numbers, exact purity of all peptides, solvents, and storage conditions are given in Table S1. The amino acid composition of all Aβ peptides corresponded to the human WT sequence. Aβ_1-42_ peptides were purchased from Anaspec/MoBiTec, Peptides&Elephants, and Sigma-Aldrich or synthesized by Synpeptide. All Aβ_1-42_ peptides were dissolved at 30 μM in C1 assay buffer (130 mM NaCl, 10 mM Hepes, 5 mM KCl, 2 mM CaCl_2_, pH 7.2, all purchased from Carl Roth). To fully dissolve the peptides in C1, they were placed in an ultrasonic bath for usually 5 to 10 min at room temperature until all precipitates had vanished. Peptides dissolved in an aqueous solution were then aliquoted for single use, immediately stored at −20 °C, and used up within 6 weeks because longer storage may affect the results. If not otherwise stated, experiments were performed with Aβ_1-42_ dissolved in C1. For selected experiments (Table S1) where we investigated the effects of different solvents Aβ_1-42_ from Anaspec/MoBiTec, P&E, and Synpeptide were dissolved at 5 mM in DMSO (Sigma-Aldrich), Aβ_1-42_ from P&E was additionally dissolved at 30 μM in HBSS (140 mM NaCl, 5 mM KCl, 1 mM CaCl_2_, 0.4 mM Magnesium Sulfate Heptahydrate, 0.5 mM Magnesium Chloride Hexahydrate, 0.3 mM Sodium Phosphate Dihydrate, 0.4 mM Potassium Phosphate, 4 mM Sodium bicarbonate, pH 7.2, all purchased from Carl Roth) or Tris–NaCl (50 mM Tris, 150 mM NaCl, pH 7.2). Peptides in HBSS or Tris–NaCl were dissolved in an ultrasonic bath as described above. HFIP-pretreated Aβ_1-42_ (in the main text referred to as Anaspec-HFIP) was purchased as AggreSure Aβ_1-42_ from Anaspec/MoBiTec and dissolved at 5 mM in DMSO for calcium imaging experiments or in Tris–NaCl (50 mM Tris, 150 mM NaCl, pH 7.2) for aggregation assays. Aβ_1-40_, Aβ_1-10_, and Aβ_1-16_ were purchased from Anaspec/MoBiTec. The Aβ_17-40_ peptide used throughout the main text was purchased from Anaspec/MoBiTec and validated with Aβ_17-40_ peptides from Sigma-Aldrich and Synpeptide. Aβ_11-40_ was obtained from Peptides&Elephants. Aβ_1-10_ and Aβ_1-16_ were dissolved in C1 as a 1 mM stock solution and Aβ_1-40_ at 30 μM as described above. Due to their high hydrophobicity, Aβ_11-40_ and Aβ_17-40_ were dissolved in DMSO at 5 mM. Tert-Boc-FLFL (tBoc2) was purchased from Bachem and dissolved in DMSO at 30 mM. f-MLFYFS (Psy-SP6) and f-MAMKKL (Sal-SP4) were obtained from VCPBIO and dissolved at 10 mM in DMSO (Psy-SP6) or at 1 mM in C1 (SP4), respectively. WKWMVm-NH_2_ and WKWMVm-CHO were purchased from Proteogenix and VCPBIO, respectively, and dissolved as 1 mM stock solutions in C1. fMLF was purchased from Sigma-Aldrich and also dissolved at 1 mM in C1. All peptides were thawed at room temperature approximately 1 h before each functional experiment, as this time was needed to prepare the compound plates for each measurement.

### Cell culture and transient transfections

HEK293T cells (ATCC) were cultured in Dulbecco’s Modified Eagle’s medium (DMEM, Biowest) supplemented with 10% (v/v) heat-inactivated fetal calf serum (FCS, Pan Biotech), 1 unit/ml penicillin-streptomycin (Biowest), and 2 mM L-glutamine (Biowest) until 80% confluence. For transfection, approximately 2000 cells were seeded in each well of poly-D-lysine–coated (PDL) (10 μg/ml in PBS, Sigma) black optical 96-well μCLEAR-plates (Greiner Bio-One). Cells were transfected after 24 h using jetPEI (Polyplus-transfection SA) with 0.125 μg of DNA plasmids encoding the respective receptors and equal amounts of a plasmid encoding the G protein subunit Gα_16_ since this subunit is needed to trigger FPR-dependent calcium signals in HEK293 cells (50, 70). The medium of transfected cells was changed 24 h after transfection. For mock transfections, the proportion of the FPR-containing plasmids were substituted by an empty pcDNA3.1 vector.

U87 MG cells (CLS) were cultured in Minimum Essential medium (Biowest) supplemented with 10% (v/v) heat-inactivated FCS (Pan Biotech), 1 unit/ml penicillin-streptomycin (Biowest), 2 mM L-glutamine (Biowest), and 1 unit/ml non-essential amino acids (Biowest) until 50 to 60% confluence. For calcium imaging and immunostaining, on average 2500 cells were seeded in each well of PDL-coated (10 μg/ml in PBS, Sigma) black optical 96-well μCLEAR-plates (Greiner Bio-One).

### Calcium imaging

Cell population responses of transfected HEK293T cells and U87 cells were recorded using a Flexstation III microplate reader (Molecular Devices). Briefly, cells were incubated with 2 μM Calbryte 520 AM (AAC Bioquest) for 2 h at room temperature in C1 assay buffer with 5 mM glucose (Carl Roth). Before each experiment, cells were rinsed three times with C1. Experiments with HEK293T cells were conducted 48 h after transfection, while experiments with U87 cells were performed 24 h after seeding. Acquisition of baseline fluorescence was performed for 25 s before ligand application, and cell population response was measured for 125 s after application. Responsiveness of cells was controlled by the appropriate buffer and solvent controls and with 10 μM WKWMVm-NH_2_ or WKWMVm-CHO as those ligands are potent activators of all three human and mouse FPRs (50).

### Chemotaxis assays

Cell migration assays were performed and analyzed with an IncuCyte S3 Life Cell Imaging System (EssenBio Science). Briefly, U87 cells were harvested and resuspended in Minimum Essential medium (Biowest) supplemented with 0.5% (v/v) heat-inactivated FCS (Pan Biotech), 1 unit/ml penicillin-streptomycin (Biowest), 2 mM L-glutamine (Biowest), and 1 unit/ml non-essential amino acids (Biowest). Approximately, 1000 cells were placed into the top chamber of each well of a 96-well ClearView Plate (EssenBioscience). Chemoattractants were mixed in 200 μl medium and then placed in the corresponding bottom chambers. Image acquisition was performed hourly for 24 h on both sides of a membrane separating the top and bottom chambers. Subsequent analysis was performed using the IncuCyte S3 software.

### Immunostaining

Approximately, 2000 HEK293T cells or 3500 U87 cells were seeded in each well of a PDL-coated black optical 96-well μCLEAR-plate (Greiner Bio-One). Transient transfection of HEK293T cells was performed as described above. Twenty four hours after transfection, cells were fixated with 4% [v/v] methanol-free paraformaldehyde (Polyscience Inc) in PBS for 30 min at RT and afterward rinsed with PBS. After blocking with 5% [v/v] FCS in PBS for 30 min at RT, primary antibodies diluted in blocking solution were applied to the cells and incubated over night at 4 °C. Hereby, monoclonal antibodies for hFPR1 (R&D Systems, MAB3744, 1 μg/ml), hFPR2 (Santa Cruz Biotechnology, sc-57141, 0.2 μg/ml), and hFPR3 (R&D Systems, MAB3896, 1 μg/ml) were used. Cells were rinsed three times with PBS and subsequently treated with 2 μg/ml polyclonal alpaca anti-mouse antibody conjugated with Alexa Fluor 568 (Invitrogen) and 2 μM Hoechst 33342 (Thermo Fisher) for 60 min at RT. Image acquisition was performed with a Molecular Devices ImageXpress Micro confocal microscope and analyzed using MetaXpress software (Molecular Devices).

### PCR analysis

Total RNA was isolated using AnalytikJena innuPREP RNA Kit according to the manufacturer’s protocol. Reverse transcription was carried out using 30 ng of total RNA and Superscript II Reverse Transcriptase (ThermoFisher Scientific). Initial reverse transcription polymerase chain reaction experiments were performed using DreamTaq DNA polymerase (ThermoFisher Scientific) with primers (Sigma-Aldrich) comprising the full coding region with complementary DNA (cDNA) obtained from 0.3 ng total RNA in a total reaction volume of 20 μl. PCR conditions were as follows: 95 °C for 3 min, 35 cycles at 95 °C for 30 s, 64 °C for 30 s, and 72 °C for 60 s, followed by a final extension of 72 °C for 10 min. qPCRs were performed with the Biozym Blue S'Green qPCR Kit according to the MIQE guidelines (71). Reverse transcription quantitative polymerase chain reaction reactions were carried out with cDNA obtained from 0.15 ng total RNA as duplicates in 20 μl total reaction volume. PCR conditions were as follows: 95 °C for 3 min, 32 cycles at 95 °C for 5 s, 66 °C for 10 s, and 66 °C for 10 s. Representative samples of all PCR products were assessed by gel electrophoresis and sequencing for their quality. Absolute quantification of DNA copies was calculated according to the specific standard curves supplemented in Figure S6. For qPCR for calibration curves, a dilution series of a sequenced and purified PCR product in 2 ng/μl yeast tRNA (Sigma-Aldrich) was used. In addition, relative quantifications for a house keeping gene were performed using GAPDH. All PCRs were performed in a TOPical T-Gradient thermocycler (Biometra). Primer sequences are given in Table S2.

### ThT aggregation assay

For the experiments in C1 buffer, Aβ_1-42_ peptides were dissolved as 30 μM stock solutions For experiments with different buffers, Aβ_1-42_ from P&E was dissolved either at 5 mM in DMSO or at 30 μM in HBSS or Tris–NaCl as described above. HFIP-treated Aβ_1-42_ was dissolved at 5 mM in DMSO and measured in C1 with a final concentration of 0.2% [v/v] DMSO. ThT (Sigma-Aldrich) stock solutions were dissolved at 1 mM in the respective buffer, sterile filtered with 0.2 μM pore membranes, and stored at −20 °C. Aβ peptides were combined with ThT stocks to produce working solutions with final concentrations of 22.5 μM Aβ_1-42_ and 250 μM ThT in the respective buffer. Fluorescence measurements were carried out as 100 μl triplicates in black optical 384-well μCLEAR-plates (Greiner Bio-One) at 37 °C with excitation at 440 nm and emission at 484 nm in a FlexStation III microplate reader within 15 min after thawing of the peptides. Signals were measured in intervals of 60 s for 120 min and were shaken for 3 s before each read.

### Binding assay

Approximately, 2000 HEK293T cells were seeded in each well of a PDL-coated black optical 96-well plate and transfected as described above. Forty eight hours after transfection, cells were treated with 1 μM of FITC-labeled WKWVm-NH_2_ and 20 μM Hoechst 33342 diluted in DMEM and incubated for 30 min at 37 °C and 5% CO_2_. Cells were then rinsed ten times with C1. Image acquisition was performed with a Molecular Devices ImageXpress Micro confocal microscope and analyzed using MetaXpress software (Molecular Devices).

### CD spectroscopy

Thirty micromolar of Aβ_1-42_ dissolved in C1 were sonicated for 10 min before CD measurement. CD spectra were recorded (JASCO J-1500 spectrometer) in a 1 mm High Precision Cell (Hellma Analytics). Data were processed in Spectra Analysis by JASCO and were plotted by Origin.

### Transmission electron microscopy

Thirty micromolar of Aβ_1-42_ peptide solution was first incubated for 24 h and then deposited on Formvar/carbon-film–coated copper grids (Plano GmbH). Samples were then stained with 4% uranyl acetate. Subsequently, TEM images were acquired (JEOL 1400 Transmission Electron Microscope) and then processed in ImageJ.

### Statistical methodology

Statistical significances were calculated using either the unpaired student’s t-test with assumption of Gaussian distribution or one-way ANOVA with Dunnett’s or Tukey’s multiple comparisons post hoc analysis. Calculations were performed using GraphPad Prism 9.2.

## Data availability

The authors confirm that the data supporting the findings of this study are available within the article and its supporting information.

## Supporting information

This article contains supporting information.

## Conflict of interest

The authors declare that they have no conflicts of interest with the contents of this article.

## Figures and Tables

**Supplemental Figure S8:**
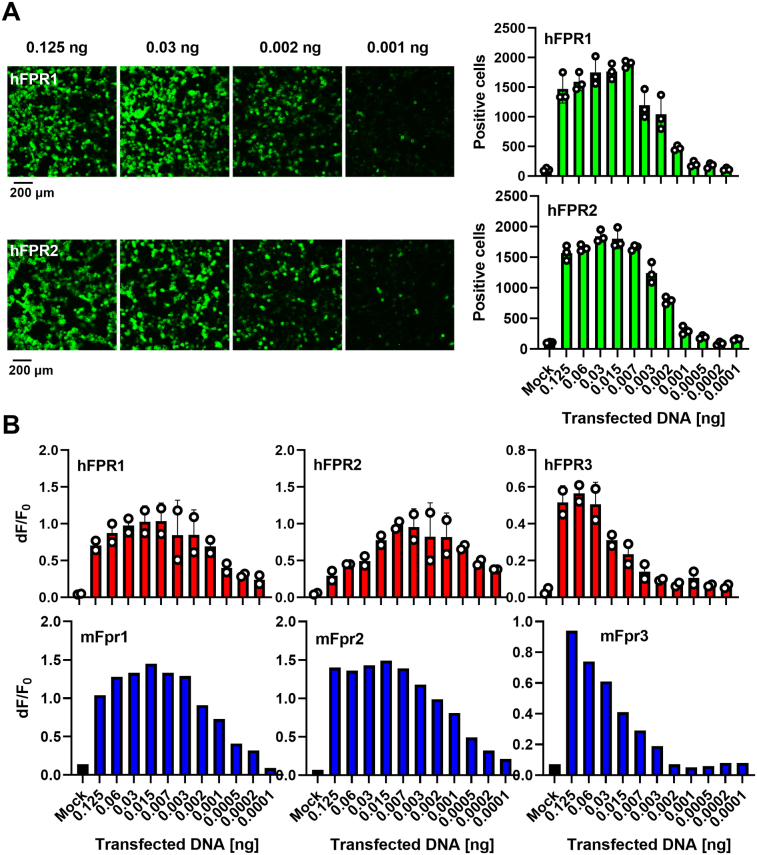
Evidence for similar cell surface expression and calcium responses of FPR1 and FPR2 in HEK293T cells. A, HEK293T cells were transfected with varying amounts of plasmid DNA for hFPR1 or hFPR2 and subsequently treated with 1 μM of FITC-labeled WKWVm-NH2 to monitor their cell surface expression. Left: representative images of treated cells transfected with different DNA amounts Right: quantitative analysis of positively stained cells. Note that cell surface expression of both receptors upon dilution is reduced in a similar manure. Error bars, S.D. n=1, N=3 B, Mean Ca2+ peak responses of HEK293T cells transfected with decreasing amounts of human (red) or mouse (blue) FPR plasmid DNA. FPR1 and 2 of each species were treated with 1 μM of WKWVm-NH2 and FPR3-transfected cells with 30 μM of WKWVm-CHO. Note that the amount of FPR 1 and 2 DNA can be diminished by more than 10fold without a strong reduction of the signal amplitudes.

## Supporting information

Supplemental Table S1 Details of all used Aβ peptides. The table details the manufacturers, catalog and lot numbers and purity of all Aβ peptides that were used within this work. CaI: Calcium Imaging, ChT: Chemotaxis, ThT: ThT Aggregation Assay. Buffer compositions are detailed in the Experimental procedures.

Supplemental Table S2 Details of all primers used for PCR experiments and Reverse Transcriptase. Accession numbers and primer sequences which were used for RT-PCR, RT-qPCR and during Reverse Transcriptase.

## References

[1] AlzheimerAssociation (2019). 2019 Alzheimer’s disease facts and figures. Alzheimer’s Dement..

[2] Long J.M., Holtzman D.M. (2019). Alzheimer disease: an update on pathobiology and treatment strategies. Cell.

[3] Masters C.L., Simms G., Weinman N.A., Multhaup G., McDonald B.L., Beyreuther K. (1985). Amyloid plaque core protein in Alzheimer disease and Down syndrome. Proc. Natl. Acad. Sci. U. S. A..

[4] Grundke-Iqbal I., Iqbal K., Tung Y.C., Quinlan M., Wisniewski H.M., Binder L.I. (1986). Abnormal phosphorylation of the microtubule-associated protein tau (tau) in Alzheimer cytoskeletal pathology. Proc. Natl. Acad. Sci. U. S. A..

[5] Kosik K.S., Joachim C.L., Selkoe D.J. (1986). Microtubule-associated protein tau (τ) is a major antigenic component of paired helical filaments in Alzheimer disease. Proc. Natl. Acad. Sci. U. S. A..

[6] Zetterberg H., Blennow K., Hanse E. (2010). Amyloid β and APP as biomarkers for Alzheimer’s disease. Exp. Gerontol..

[7] Masters C.L., Selkoe D.J. (2012). Biochemistry of amyloid β-protein and amyloid deposits in Alzheimer disease. Cold Spring Harb. Perspect. Med..

[8] Akiyama H., Barger S., Barnum S., Bradt B., Bauer J., Cole G.M. (2000). Inflammation and Alzheimer’s disease. Neurobiol. Aging.

[9] Ries M., Sastre M. (2016). Mechanisms of Aβ clearance and degradation by glial cells. Front. Aging Neurosci..

[10] Haass C., Kaether C., Thinakaran G., Sisodia S. (2012). Trafficking and proteolytic processing of APP. Cold Spring Harb. Perspect. Med..

[11] Steiner H., Fukumori A., Tagami S., Okochi M. (2018). Making the final cut: pathogenic amyloid-β peptide generation by γ-secretase. Cell Stress.

[12] Busch L., Vieten S., Brödel S., Endres K., Bufe B. (2022). Emerging contributions of formyl peptide receptors to neurodegenerative diseases. Biol. Chem..

[13] Shea D., Hsu C.C., Bi T.M., Paranjapye N., Childers M.C., Cochran J. (2019). α-Sheet secondary structure in amyloid β-peptide drives aggregation and toxicity in Alzheimer’s disease. Proc. Natl. Acad. Sci. U. S. A..

[14] Leng F., Edison P. (2021). Neuroinflammation and microglial activation in Alzheimer disease: Where do we go from here?. Nat. Rev. Neurol..

[15] Guzman-Martinez L., Maccioni R.B., Andrade V., Navarrete L.P., Pastor M.G., Ramos-Escobar N. (2019). Neuroinflammation as a common feature of neurodegenerative disorders. Front. Pharmacol..

[16] Haass C., Selkoe D.J. (2007). Soluble protein oligomers in neurodegeneration: lessons from the Alzheimer’s amyloid β-peptide. Nat. Rev. Mol. Cell Biol..

[17] Nimmerjahn A., Kirchhoff F., Helmchen F. (2005). Neuroscience: resting microglial cells are highly dynamic surveillants of brain parenchyma *in vivo*. Science.

[18] Rodríguez-Gómez J.A., Kavanagh E., Engskog-Vlachos P., Engskog M.K.R., Herrera A.J., Espinosa-Oliva A.M. (2020). Microglia: agents of the CNS pro-inflammatory response. Cells.

[19] Tolar M., Hey J., Power A., Abushakra S. (2021). Neurotoxic soluble amyloid oligomers drive Alzheimer’s pathogenesis and represent a clinically validated target for slowing disease progression. Int. J. Mol. Sci..

[20] Dahlgren K.N., Manelli A.M., Stine W.B., Baker L.K., Krafft G.A., LaDu M.J. (2002). Oligomeric and fibrillar species of amyloid-β peptides differentially affect neuronal viability. J. Biol. Chem..

[21] Wang J., Jackson M.F., Xie Y.F. (2016). Glia and TRPM2 channels in plasticity of central nervous system and Alzheimer’s diseases. Neural Plast..

[22] Wilkinson K., Khoury El (2012). J. Microglial scavenger receptors and their roles in the pathogenesis of Alzheimer’s disease. Int. J. Alzheimers. Dis..

[23] Fiebich B.L., Batista C.R.A., Saliba S.W., Yousif N.M., de Oliveira A.C.P. (2018). Role of microglia TLRs in neurodegeneration. Front. Cell. Neurosci..

[24] Heneka M.T., Kummer M.P., Stutz A., Delekate A., Schwartz S., Vieira-Saecker A. (2012). NLRP3 is activated in Alzheimer’s disease and contributes to pathology in APP/PS1 mice. Nature.

[25] Doens D., Fernández P.L. (2014). Microglia receptors and their implications in the response to amyloid β for Alzheimer’s disease pathogenesis. J. Neuroinflammation.

[26] Ye R.D., Boulay F., Wang J.M., Dahlgren C., Gerard C., Parmentier M. (2009). International union of basic and clinical pharmacology. LXXIII. Nomenclature for the formyl peptide receptor (FPR) family. Pharmacol. Rev..

[27] Bloes D.A., Kretschmer D., Peschel A. (2015). Enemy attraction: bacterial agonists for leukocyte chemotaxis receptors. Nat. Rev. Microbiol..

[28] Dahlgren C., Gabl M., Holdfeldt A., Winther M., Forsman H. (2016). Basic characteristics of the neutrophil receptors that recognize formylated peptides, a danger-associated molecular pattern generated by bacteria and mitochondria. Biochem. Pharmacol..

[29] Migeotte I., Communi D., Parmentier M. (2006). Formyl peptide receptors: a promiscuous subfamily of G protein-coupled receptors controlling immune responses. Cytokine Growth Factor Rev..

[30] Bufe B., Zufall F. (2016). The sensing of bacteria: emerging principles for the detection of signal sequences by formyl peptide receptors. Biomol. Concepts.

[31] Brandenburg L.O., Konrad M., Wruck C.J., Koch T., Lucius R., Pufe T. (2010). Functional and physical interactions between formyl-peptide-receptors and scavenger receptor MARCO and their involvement in amyloid beta 1-42-induced signal transduction in glial cells. J. Neurochem..

[32] Becker E.L., Forouhar F.A., Grunnet M.L., Boulay F., Tardif M., Bormann B.J. (1998). Broad immunocytochemical localization of the formylpeptide receptor in human organs, tissues, and cells. Cell Tissue Res.

[33] Cattaneo F., Guerra G., Ammendola R. (2010). Expression and signaling of formyl-peptide receptors in the brain. Neurochem. Res..

[34] Colucci M., Stefanucci A., Mollica A., Aloisi A.M., Maione F., Pieretti S. (2022). New insights on formyl peptide receptor type 2 involvement in nociceptive processes in the spinal cord. Life (Basel).

[35] Slowik A., Merres J., Elfgen A., Jansen S., Mohr F., Wruck C.J. (2012). Involvement of formyl peptide receptors in receptor for advanced glycation end products (RAGE) - and amyloid beta 1-42-induced signal transduction in glial cells. Mol. Neurodegener..

[36] Le Y., Gong W., Tiffany H.L., Tumanov A., Nedospasov S., Shen W. (2001). Amyloid B 42 activates a G-protein-coupled chemoattractant. J. Neurosci..

[37] Brandenburg L.O., Konrad M., Wruck C., Koch T., Pufe T., Lucius R. (2008). Involvement of formyl-peptide-receptor-like-1 and phospholipase D in the internalization and signal transduction of amyloid beta 1-42 in glial cells. Neuroscience.

[38] Tiffany H.L., Lavigne M.C., Cui Y.H., Wang J.M., Leto T.L., Gao J.L. (2001). Amyloid-β induces chemotaxis and oxidant stress by acting at formylpeptide receptor 2, a G protein-coupled receptor expressed in phagocytes and brain. J. Biol. Chem..

[39] Lorton D., Schaller J., Lala A., De Nardin E. (2000). Chemotactic-like receptors and Aβ peptide induced responses in Alzheimer’s disease. Neurobiol. Aging.

[40] Cui Y., Le Y., Yazawa H., Gong W., Wang J.M. (2002). Potential role of the formyl peptide receptor-like 1 (FPRL1) in inflammatory aspects of Alzheimer’s disease. J. Leukoc. Biol..

[41] Wickstead E.S., Karim H.A., Manuel R.E., Biggs C.S., Getting S.J., McArthur S. (2020). Reversal of β-amyloid-induced microglial toxicity in vitro by activation of Fpr2/3. Oxid. Med. Cell. Longev..

[42] Le Y., Gong W., Tiffany H.L., Tumanov A., Nedospasov S., Shen W. (2001). Amyloid (beta)42 activates a G-protein-coupled chemoattractant receptor, FPR-like-1. J. Neurosci..

[43] Yazawa H., Yu Z.-X., Takeda K., Le Y., Gong W., Ferrans V.J. (2001). β Amyloid peptide (Aβ 42 ) is internalized via the G-protein-coupled receptor FPRL1 and forms fibrillar aggregates in macrophages 1. FASEB J..

[44] Zhang S., Gong H., Ge Y., Ye R.D. (2020). Biased allosteric modulation of formyl peptide receptor 2 leads to distinct receptor conformational states for pro- and anti-inflammatory signaling. Pharmacol. Res..

[45] Kong Y., Ruan L., Qian L., Liu X., Le Y. (2010). Norepinephrine promotes microglia to uptake and degrade amyloid β peptide through upregulation of mouse formyl peptide receptor 2 and induction of insulin-degrading enzyme. J. Neurosci..

[46] Schröder N., Schaffrath A., Welter J.A., Putzka T., Griep A., Ziegler P. (2020). Inhibition of formyl peptide receptors improves the outcome in a mouse model of Alzheimer disease. J. Neuroinflammation.

[47] Gao J.L., Chen H., Filie J.D., Kozak C.A., Murphy P.M. (1998). Differential expansion of the N-formylpeptide receptor gene cluster in human and mouse. Genomics.

[48] Liberles S.D., Horowitz L.F., Kuang D., Contos J.J., Wilson K.L., Siltberg-Liberles J. (2009). Formyl peptide receptors are candidate chemosensory receptors in the vomeronasal organ. Proc. Natl. Acad. Sci. U. S. A..

[49] Boillat M., Carleton A., Rodriguez I. (2021). From immune to olfactory expression: Neofunctionalization of formyl peptide receptors. Cell Tissue Res.

[50] Bufe B., Schumann T., Zufall F. (2012). Formyl peptide receptors from immune and vomeronasal system exhibit distinct agonist properties. J. Biol. Chem..

[51] Bufe B., Teuchert Y., Schmid A., Pyrski M., Pérez-Gómez A., Eisenbeis J. (2019). Bacterial MgrB peptide activates chemoreceptor Fpr3 in mouse accessory olfactory system and drives avoidance behaviour. Nat. Commun..

[52] Nichols M.R., Moss M.A., Reed D.K., Hoh J.H., Rosenberry T.L. (2005). Amyloid-beta aggregates formed at polar-nonpolar interfaces differ from amyloid-beta protofibrils produced in aqueous buffers. Microsc. Res. Tech..

[53] Szczepanik A.M., Rampe D., Ringheim G.E. (2001). Amyloid-β peptide fragments p3 and p4 induce pro-inflammatory cytokine and chemokine production *in vitro* and *in vivo*. J. Neurochem..

[54] Näslund J., Schierhorn A., Hellman U., Lannfelt L., Roses A.D., Tjernberg L.O. (1994). Relative abundance of Alzheimer Aβ amyloid peptide variants in Alzheimer disease and normal aging. Proc. Natl. Acad. Sci. U. S. A..

[55] Kuhn A.J., Raskatov J. (2020). Is the p3 peptide (Aβ17-40, Aβ17-42) relevant to the pathology of Alzheimer’s disease?. J Alzheimers Dis.

[56] Boer J.C., van Marion D.M., Joseph J.V., Kliphuis N.M., Timmer-Bosscha H., van Strijp J.A. (2015). Microenvironment involved in FPR1 expression by human glioblastomas. J. Neurooncol..

[57] Boer J.C., Domanska U.M., Timmer-Bosscha H., Boer I.G., de Haas C.J., Joseph J.V. (2013). Inhibition of formyl peptide receptor in high-grade astrocytoma by CHemotaxis Inhibitory Protein of S. aureus. Br. J. Cancer.

[58] Stempel H., Jung M., Pérez-Gómez A., Leinders-Zufall T., Zufall F., Bufe B. (2016). Strain-specific loss of Formyl peptide receptor 3 in the murine vomeronasal and immune systems. J. Biol. Chem..

[59] Calvello R., Cianciulli A., Porro C., Moda P., De Nuccio F., Nicolardi G. (2021). Formyl peptide receptor (Fpr)1 modulation by resveratrol in an lps-induced neuroinflammatory animal model. Nutrients.

[60] Wickstead E.S., Irving M.A., Getting S.J., McArthur S. (2021). Exploiting formyl peptide receptor 2 to promote microglial resolution: a new approach to Alzheimer’s disease treatment. FEBS J..

[61] Walther A., Riehemann K., Gerke V. (2000). A novel ligand of the formyl peptide receptor: annexin I regulates neutrophil extravasation by interacting with the FPR. Mol. Cell.

[62] Filep J.G. (2013). Biasing the lipoxin A4/formyl peptide receptor 2 pushes inflammatory resolution. Proc. Natl. Acad. Sci. U. S. A..

[63] Serhan C.N., Chiang N., Van Dyke T.E. (2008). Resolving inflammation: Dual anti-inflammatory and pro-resolution lipid mediators. Nat. Rev. Immunol..

[64] Gröper J., König G.M., Kostenis E., Gerke V., Raabe C.A., Rescher U. (2020). Exploring biased agonism at FPR1 as a means to encode danger sensing. Cells.

[65] Seubert P., Vigo-Pelfrey C., Esch F., Lee M., Dovey H., Davis D. (1992). Isolation and quantification of soluble Alzheimer’s beta-peptide from biological fluids. Nature.

[66] Barritt J.D., Younan N.D., Viles J.H. (2017). N-terminally truncated amyloid-β (11-40/42) cofibrillizes with its full-length counterpart: implications for Alzheimer’s disease. Angew. Chem. Int. Ed. Engl..

[67] Barritt J.D., Younan N.D., Viles J.H. (2017). N-Terminally truncated amyloid-β (11 – 40/42) cofibrillizes with its full-length counterpart: implications for Alzheimer’s disease. Angew. Chem..

[68] Giulian D., Haverkamp L.J., Yu J.H., Karshin W., Tom D., Li J. (1996). Specific domains of beta-amyloid from Alzheimer plaque elicit neuron killing in human microglia. J. Neurosci..

[69] Heurtaux T., Michelucci A., Losciuto S., Gallotti C., Felten P., Dorban G. (2010). Microglial activation depends on beta-amyloid conformation: role of the formylpeptide receptor 2. J. Neurochem..

[70] Offermanns S., Simon M.I. (1995). G alpha 15 and G alpha 16 couple a wide variety of receptors to phospholipase C. J. Biol. Chem..

[71] Bustin S.A., Benes V., Garson J.A., Hellemans J., Huggett J., Kubista M. (2009). The MIQE guidelines: minimum information for publication of quantitative real-time PCR experiments. Clin. Chem..

[72] Humphrey W., Dalke A., Schulten K. (1996). Vmd: visual molecular dynamics. J. Mol. Graph..

[73] Crescenzi O., Tomaselli S., Guerrini R., Salvadori S., D'Ursi A.M., Temussi P.A. (2002). Solution structure of the Alzheimer amyloid β-peptide (1–42) in an apolar microenvironment. Eur. J. Biochem..

