# Supplementary material for: Amyloid beta and its naturally occurring N-terminal variants are potent activators of human and mouse formyl peptide receptor 1
Source: J Biol Chem. 2022 Oct 27;298(12):102642. doi: 10.1016/j.jbc.2022.102642 (PMC9694488; doi:10.1016/j.jbc.2022.102642)
Supplement: Supplemental Table S1 — Details of all used Aβ peptides. The table details the manufacturers, catalog and lot numbers and purity of all Aβ peptides that were used within this work. CaI: Calcium Imaging, ChT: Chemotaxis, ThT: ThT Aggregation Assay. Buffer compositions are detailed in the Experimental procedures. [file mmc1.docx]

| Name | Manufacturer | Catalog# | Lot# | Purity | Stock conc.  and solvent | Max. End conc.  in Assay |
| --- | --- | --- | --- | --- | --- | --- |
| Aβ 1-10 | Anaspec | AS-64478 | 2055677 | 96% | 1 mM / C1 | CaI: 10 µM |
| Aβ 1-16 | Anaspec | AS-24225 | n.n. | 96% | 1 mM / C1 | CaI: 10 µM |
| Aβ 1-40 | Anaspec | AS-24235 | 2158441 | 96% | 30 µM / C1 | CaI: 10 µM |
| Aβ 1-40 | Anaspec | AS-24235 | 2056942 | 95% | 30 µM / C1 | CaI: 10 µM |
| Aβ 1-42 | Peptides &  elephants | EP10060 | 2206R05 | 95.50% | 5 mM / DMSO  30 µM / C1  30 µM / Tris-NaCl  30 µM / HBSS | CaI: 10 µM  ChT: 10 µM  ThT: 22.5 µM |
| Aβ 1-42 | Anaspec | AS-20276 | 2155092 | 95% | 5 mM / DMSO  30 µM / C1 | CaI: 10 µM  ThT: 22.5 µM |
| Aβ 1-42 | Synpeptide | custom synthesis | | 95% | 5 mM / DMSO  30 µM / C1 | CaI: 10 µM  ChT: 10 µM  ThT: 22.5 µM |
| Aβ 1-42 | Sigma-Aldrich | PP69 | 3760648 | 95% | 30 µM / C1 | CaI: 10 µM  ThT: 22.5 µM |
| Aβ 11-40 | Peptides &  elephants | EP10013 | 0206U02 | 95.10% | 5 mM DMSO | CaI: 5 µM  ChT: 1 µM |
| Aβ 17-40 | Anaspec | AS-22813 | 2156695 | 98% | 5 mM DMSO | CaI: 5 µM  ChT: 5 µM |
| Aβ 17-40 | Synpeptide | custom synthesis | | 95% | 5 mM DMSO | CaI: 5 µM |
| Aβ 17-40 | Sigma-Aldrich | A4848 | 117K1109 | 91% | 5 mM DMSO | CaI: 5 µM |
