# Supplementary material for: Amyloid beta and its naturally occurring N-terminal variants are potent activators of human and mouse formyl peptide receptor 1
Source: J Biol Chem. 2022 Oct 27;298(12):102642. doi: 10.1016/j.jbc.2022.102642 (PMC9694488; doi:10.1016/j.jbc.2022.102642)
Supplement: Supplemental Table S2 — Details of all primers used for PCR experiments and Reverse Transcriptase. Accession numbers and primer sequences which were used for RT-PCR, RT-qPCR and during Reverse Transcriptase. [file mmc2.docx]

| Accession No. | Gene | Official Full Name | Oligonucleotide sequence |
| --- | --- | --- | --- |
| Reverse Transcription primers | | | |
| n.n. | n.n. | CDS (Smart) | 5-AGCAGTGGTAACAACGCAGAGTA  CTTTTTTTTTTTTTTTTTTTTTTTTTTTTTTVN |
| n.n. | n.n. | SMART II | 5- AAGCAGTGGTAACAACGCAGAGTA  CGCGGG |
| RT-PCR Primer | | | |
|  |  |  |  |
| NM_002029.4 | hFPR1 | Homo sapiens formyl peptide receptor 1 (FPR1), transcript variant 2, mRNA | 5-AAAGAATTCAAGCTTCCTGCAGGCGC  CACCATGGAGACAAATTCCTCTCTCCC-3  5-TTGGATATCGCGGCCGCAAGAGCTCA CTTTGCCTGTAACTCCACCTCTGC-3 |
| NM_001005738 | hFPR2 | Homo sapiens formyl peptide receptor 2 (FPR2), transcript variant 2, mRNA | 5-AAAGAATTCAAGCTTCCTGCAGGCGCC  ACCATGGAAACCAACTTCTCCACTCCTC-3  5-TTGGATATCGCGGCCGCAAGAGCTCA  CATTGCCTGTAACTCAGTCTCTGCA-3 |
| NM_002030.5 | hFPR3 | Homo sapiens formyl peptide receptor 3 | 5-AAAGAATTCAAGCTTCCTGCAGGCGCC  ACC ATGGAAACCAACTTCTCCATTCCT-3  5-TTGGATATCGCGGCCGCAAGAGCTCA  CATTGCTTGTAACTCCGTCTCCTC-3 |
|  |  |  |  |
| RT-qPCR primers | | | |
| NM_002029.4 | hFPR1 | Homo sapiens formyl peptide receptor 1 (FPR1), transcript variant 2, mRNA | 5-GGGTCCTCTCCTTTGTCGCAGCA-3  5-GGCGGGAAGGGCGTGGATCA-3 |
| NM_001005738 | hFPR2 | Homo sapiens formyl peptide receptor 2 (FPR2), transcript variant 2, mRNA | 5-GCTTGCCGATGTCCATTGTTGCCA-3  5-GGCCAGGGAGCTCGTTGGGT-3 |
| NM_002030.5 | hFPR3 | Homo sapiens formyl peptide receptor 3 | 5-TCAGCGTGCCTATGTCCATCA-3  5-ACCACAGCAGCGAAGACACG-3 |
| NM_002046.7 | hGAPDH | Homo sapiens glyceraldehyde-3-phosphate dehydrogenase | 5-GAAGGTGAAGGTCGGAGTC-3  5-GAAGATGGTGATGGGATTTC-3 |
